# Contributions of leaf distribution and leaf functions to photosynthesis and water-use efficiency from leaf to canopy in apple: A comparison of interstocks and cultivars

**DOI:** 10.3389/fpls.2023.1117051

**Published:** 2023-04-14

**Authors:** Xiaoyun Zhang, Weiwei Yang, Muhammad Mobeen Tahir, Xilong Chen, Marc Saudreau, Dong Zhang, Evelyne Costes

**Affiliations:** ^1^College of Agriculture, The Key Laboratory of Special Fruits and Vegetables Cultivation Physiology and Germplasm Resources Utilization in Xinjiang Production and Construction Group, Shihezi University, Shihezi, Xinjiang, China; ^2^College of Horticulture, Northwest A&F University, Yangling, Shaanxi, China; ^3^Université Clermont Auvergne, INRAE, PIAF, Clermont-Ferrand, France; ^4^UMR AGAP Institute, University of Montpellier, INRAE, Institut Agro, CIRAD, Equipe ‘Architecture et Floraison des Especes Fruiteres’, Montpellier, France

**Keywords:** *Malus domestica*, RATP model, stomatal conductance, leaf functional traits, dwarfing interstocks, virtual scenario, 3D architecture-based model

## Abstract

Grafting has been widely used in horticulture to induce dwarfing and avoid stress-derived limitations on plant growth and yield by affecting plant architecture and leaf functions. However, the respective effects on plant photosynthesis and water use efficiency (*WUE*) of leaf distribution and functions that depend on both rootstock and scion have not been fully elucidated. This study aimed to (i) clarify the scion × interstock impacts on the variability of leaf photosynthetic traits and *WUE*, and (ii) decipher the respective effects of leaf distribution and functions on canopy photosynthesis and *WUE* (*WUEc*). Leaf gas exchange over light gradients and responses to light, CO_2_, temperature, and vapor pressure deficit were measured in two apple cultivars, ‘Liquan Fuji’ (‘Fuji’) and ‘Regal Gala’ (‘Gala’), grafted onto rootstocks combined with interstocks: a vigorous (VV, ‘Qinguan’), or a dwarf one (VD, M26). The 3D architecture-based RATP model was parameterized to estimate the canopy photosynthesis rate (*A_c_*), transpiration rate (*E*_c_), and *WUEc*. Then, virtual scenarios were used to compare the relative contributions of cultivar and interstock to canopy *A*_c_, *E*_c_, and *WUE*_c_. These scenarios changed the leaf distribution and functions of either cultivar or interstock. At the leaf scale, VD trees had significantly higher leaf nitrogen per area but a lower maximum carboxylation rate and dark respiration in both cultivars. In parallel with higher leaf stomatal conductance (*g_s_*) and transpiration in VD ‘Fuji’ and similar *g_s_* in VD ‘Gala’, VD trees showed significantly lower leaf photosynthesis rate and *WUE* than VV trees. However, lower leaf photosynthetic capacities in VD trees were compensated at the canopy scale, with *A*_c_ and *WUE*_c_ for ‘Fuji’ significantly improved in VD trees under both sunny and cloudy conditions, and for ‘Gala’ significantly improved in VD trees under cloudy conditions compared with VV trees. Switching scenarios highlighted that ‘Gala’ leaf functions and distribution and VD leaf distributions enhanced *A*_c_ and *WUE*_c_ simultaneously, irrespective of weather conditions. Up-scaling leaf gas exchange to the canopy scale by utilizing 3D architecture-based modeling and reliable measurements of tree architecture and leaf functional traits provides insights to explore the influence of genetic materials and tree management practices.

## Introduction

Grafting is an ancient technique that assembles a composite plant in the form of a shoot system (the scion) grafted on a root system (the rootstock) (Fazio et al., 2015; Foster et al., 2016; Baron et al., 2019). Although the method was originally used for clonal propagation, it has been widely used in forestry and horticulture to induce dwarfing and attain resistance to disease and tolerance to abiotic stresses, such as drought, salinity, and extreme temperatures, to enhance water- and nutrient-use efficiency (Han et al., 2019; Migicovsky et al., 2019; Zhang et al., 2022a). As most dwarfing rootstocks are shallow-rooted and sensitive to stresses, grafting a dwarf interstem between the scion and stress-tolerant rootstock can take advantage of the strength of the rootstock and the dwarfing effect of the interstem, which is termed the ‘interstock’ (Di Vaio et al., 2009). This method enables the scion, interstock, and rootstock to be selected based on desired traits with the objective of maintaining the beneficial traits of all parts of the grafted plants. Thus, understanding scion–interstock/rootstock interactions will aid in improving scion or root architectural and physiological traits to increase productivity.

Fusion of the vascular systems occurs during the connection of the rootstock and scion and can be affected by both rootstock and scion genotypes (Baron et al., 2019). The root architecture is varied among rootstocks and can modify the graft vessel diameter or density in the graft union, both of which potentially affect scion water status (Tombesi et al., 2009; Albacete et al., 2015; Xu and Ediger, 2021). Several hypotheses for rootstock control of scion vigor have been proposed, involving nutrient uptake, hydraulic conductance, and hormone regulation (Webster, 1995; Solari et al., 2006; Tworkoski and Miller, 2007; Van Hooijdonk et al., 2011). These changes lead directly to changes in leaf-scale gas exchange, through which photosynthesis provides the carbon essential for tree growth and reproduction. However, inconsistency in the influence of rootstocks on leaf gas exchange reported among previous studies (Barden and Ferree, 1979; Schechter et al., 1991; Jia, 1995; Fallahi et al., 2002; Sotiropoulos, 2008; Liu et al., 2012; An et al., 2017; Fullana-Pericàsa et al., 2020; Zhou et al., 2020). In addition, tree growth and yield are associated with the sum of seasonal net canopy photosynthesis rather than the net leaf photosynthesis rate (*A*_l_) of specific leaves of the canopy (Monteith, 1977). As a result, the photosynthesis and water use response to a rootstock or interstock must be upscaled from the leaf scale to the canopy scale.

At the canopy-scale, tree architecture is central to the tradeoff between canopy carbon gain and water use by influencing leaf area spatial distribution, intra-canopy microclimate (light, temperature, CO_2_, and humidity), water transport, and carbon assimilation and allocation (Minas et al., 2018; Coupel-Ledru et al., 2022). Leaf stomatal control of photosynthesis and water loss by transpiration can interact with intra-canopy microclimate, particularly light, and result in acclimation of leaves by modifying photosynthesis related functional traits, such as dark respiration (*R*_d_), nitrogen content per area (*N_a_*), maximum rate of carboxylation (*V_cmax_*), and maximum rate of electron transport (*J_max_*) (Hikosaka, 2003; Bauerle et al., 2007; Poorter et al., 2019). In particular, the interstock/rootstock has a profound effect on tree architecture by regulating internode and shoot growth and canopy volume (Preston, 1967; Seleznyova et al., 2003; Costes and García-Villanueva, 2007; Warschefsky et al., 2016), which directly affect the intra-canopy microclimate, heterogeneity of leaf-scale gas exchange, and related leaf functional traits within the canopy, and in turn alter energy exchange with the surrounding environment and resource use efficiencies of the canopy. Given the complexity of canopy structure, the current understanding of stomatal, photosynthesis, and water-use responses to scion–rootstock combinations is limited to pot experiments conducted in controlled environments to minimize the architecture effect, and such studies are rarely performed in field environments. Moreover, such variation in leaf functional traits and the coincident intra-canopy microclimate that are influenced by interstock/rootstock or scion genotypes are rarely taken into account.

Overall, a better understanding of scion–rootstock interactions must consider tree architecture and variability in intra-canopy microclimate and leaf functional traits induced by tree architecture, which demands a more efficient and accurate three-dimensional (3D) tree architecture-based modeling approach to represent complex relationships among the above-mentioned factors. Because of the intertwined effects between tree architecture and leaf functions, and that both are assumed to depend on interstock/rootstock and scion genotypes, it is crucial to decipher their respective effects on canopy-scale photosynthesis and water use.

Apple (*Malus* × *domestica*) is suitable for exploring scion–rootstock interactions owing to its high economic value, ease of cloning, diverse genetic resources, successful application of interstocks and rootstocks, and development of 3D architecture-based models (Costes et al., 2008; Yang et al., 2021). Numerous 3D architecture-based plant models have been established to study complex relationships among tree architecture, canopy microclimate, transpiration, and carbon assimilation and allocation to screen the relative contribution of architectural and leaf functional traits to select ‘optimized’ combinations by conducting virtual experiments (Chen et al., 2014; Picheny et al., 2017; Zhu et al., 2019; Zhang et al., 2022b). However, rule-based plant architecture simulation is time-consuming and recreation of the large tree structure in the field is difficult (Zhu et al., 2019). Similarly, imaging and terrestrial light detection and ranging-based methods underestimate shoot number in apple trees and cannot detect shoots and leaves inside the canopy (Pallas et al., 2020). Alternatively, 3D tree reconstruction through combining partial 3D digitization of all leafy shoots within the canopy, allometric relationships, and random distribution of certain organ attributes is feasible to represent the large tree structure in the field (Massonnet et al., 2008; Yang et al., 2021). Thus, the radiation absorption, transpiration, and photosynthesis (RATP) model that uses 3D digitized trees as input has been successfully developed for large tree architectures to simulate the spatial distribution of radiation and leaf gas exchange within a canopy considering the canopy structure and microclimate as well as leaf physical and physiological properties (Sinoquet et al., 2001). This model has been applied to apple trees for exploring canopy water use, carbon assimilation (Massonnet et al., 2008; Yang et al., 2021), carbon allocation (Reyes et al., 2018), and within-canopy climate variability (Ngao et al., 2017; Woods et al., 2018).

In this study, we aimed to evaluate the relative impact of the variability of leaf functions and tree architecture on canopy performance in apple trees by comparing combinations of two cultivars and two interstocks. We measured leaf gas exchange and use the RATP model to determine whether there are differences among scion–rootstock combinations in photosynthetic capacities and water use at either leaf or canopy scales and in related physiological processes (*V*_cmax_, *J*_max_, and *R*_d_) in response to the environment. Making use of this modeling approach, we performed virtual experiments to decipher the effects of leaf functions and leaf distribution as modulated by interstocks or cultivars on canopy performance. We tested all possible combinations of leaf properties (at the leaf scale) and distribution (at the canopy scale) to compare and evaluate canopy photosynthetic capacity and water use efficiency (*WUE*).

## Materials and methods

### Plant materials

The experiment was conducted at an extension station of the National Apple Industry Technology System of the Agriculture Ministry of China, Fengxiang, situated at 806 m above sea level with mean annual precipitation of 532.5 mm. In winter 1998, two apple cultivars, ‘Liquan Fuji’ (hereafter ‘Fuji’) and ‘Regal Gala’ (hereafter ‘Gala’), were grafted onto a vigorous, drought-tolerant *M. micromalus* rootstock, in combination with two interstocks: *M.* × *domestica* ‘Qinguan’, a vigorous interstock (hereafter ‘VV’), and M26, a dwarf interstock (hereafter ‘VD’). All trees were trained in accordance with the spindle training system with a 3.0–3.5 m tree height and all scaffold branches tied down below horizontal (100–120° from vertical). The length of the interstocks was 30 cm, and the grafted points were 10 cm above the soil for all trees. Trees were planted 2.0 m × 3.5 m apart in a north–south orientation. Horticultural practices included regular irrigation with mini-sprinklers, standard fertilization, and phytosanitary treatments. During the growing seasons of the experiment, all trees were left unpruned but were pruned in the winter. Fruit thinning was performed each year according to professional practices to adjust the crop load within 2.42 ± 0.20 fruits per trunk cross-sectional area (*TCSA*, cm^2^). The leaf area index (LAI) was calculated as the ratio of total leaf area (estimated from the virtual canopy) to tree spacing.

### Tree measurement and 3D digitizing

The *TCSA* is a stable index of tree vigor (Strong and Azarenko, 2000) and was estimated from the trunk circumference measured with a tape for five trees during winter for each treatment and in 2011 and 2012. In addition, canopy structures were digitized after 190 DOY (day of year) when shoots growth were ceased. The spatial coordinates of the distal and proximal points of all current-year leafy shoots were collected using an electro-magnetic 3D digitizer (Polhemus; Colchester, VT, USA) and recorded with PiafDigit software (Donès et al., 2006). All shoots in the trees were classified based on their fate as apical meristems. The floral bud develops into a rosette (also termed a bourse) with non-elongated internodes at the base and an inflorescence in the terminal position (Pratt, 1988). The bourse may give rise to one or two bourse shoots that develop immediately (Lauri et al., 1995). In addition, vegetative shoots develop from vegetative buds. A threshold of 5 cm was used to discriminate long and short shoots (Costes et al., 2006). After tree digitization, at least 15 randomly selected shoots per shoot type for each treatment in each year were digitized at the leaf scale to determine leaf Euler angles (midrib azimuth and elevation, and lamina rolling around the midrib), and the angles between the leaf petiole and shoot axis according to Farque et al. (2001). All sampled shoots were transported to the laboratory to measure the shoot length with a ruler and count the leaf number. Moreover, a non-destructive leaf photo scanner was used to obtain images of all leaves from the sampled shoots. The leaf width, leaf length, and leaf area were digitally processed using ImageJ software (1.48q 1 February 2014, https://imagej.nih.gov/ij/). Each leaf was labeled with information about the year, cultivar, interstock, shoot number, and node rank. Shoot leaf area was obtained by summing the leaf areas of leaves from the same shoot. Finally, the allometric relationships between the shoot length (*SL*) and shoot leaf area (*SLA*, equation 1), between the *SL* and the shoot leaf number (*SLN*, equation 2), between the leaf length (*LL*) and leaf area (*LA*, equation 3), between *LL* and leaf width (*LW*, equation 4), and between *LL* and petiole length (*PL*, equation 5) were established.


(1) $$SLA=a_{SLA}SL+b_{SLA}$$

(2) $$SLN=a_{SLN}SL+b_{SLN}$$

(3) $$LA=a_{LA}LL^{2}$$

(4) $$LW=a_{LW}LL+b_{LW}$$

(5) $$PL=a_{PL}LL+b_{PL}$$

where *a*_i_ and *b*_i_ are coefficients, and *i* denotes the identifier (*SLA*, *SLN*, *LW*, *LA* or *PL*) for each equation.

Distributions of leaf rolling and elevation angles were obtained based on the leaf Euler angles.

### Reconstruction of a 3D virtual canopy

The spatial position and length of leafy shoots were determined by the spatial coordinates of the distal and proximal points. During reconstruction, the following principles were assumed: (1) *SLA* and *SLN* were estimated from *SL* based on equations 1 and 2, respectively; (2) all leaves attached to the same shoot were evenly distributed with an average *LA* and which was estimated as the ratio of *SLA* to *SLN*; (3) all leaves were reconstructed as plane hexagons without considering the midrib curvature and folding angle of the lamina because their effects on light interception properties are weak (Génard et al., 2000); (4) *LL* was estimated from *LA* (equation 3), and LW (equation 4) and PL (equation 5) were estimated from *LL* and were used to calibrate the hexagonal shape; (5) petiole, midrib, and shoot axes were assumed to be in the same plane defined by the shoot axis direction and the phyllotactic angle β, which was constant and equal to the average values obtained in the field; (6) petiole insertion points along a shoot were evenly segmented with an average internode length (equal to the ratio of *SL*/*SLN*), and the connection point between the petiole and leaf was computed in accordance with the shoot coordinates, β, and PL. The connection point between the petiole and leaf was used as the coordinates of the lamina origin and leaf elevation and the rolling angle was determined from the angle distribution for each shoot type, and leaf azimuth angles were distributed following a 2/5 phyllotaxy. In total, 19 trees were generated, additional details are described by Yang et al. (2016). All fitting parameters for allometric relationships, leaf elevation and rolling angle distribution, angle between the leaf petiole and shoot axis, and all virtual canopies have been deposited in the Zenodo repository (http://doi.org/10.5281/zenodo.4049858).

### Measurements of leaf gas exchange

Gas exchange was measured at the leaf scale using a portable photosynthesis system (LI-6400, Li-Cor, Lincoln, NE, USA) from 08:30 to12:30 during 217 DOY (day of year) to 225 DOY for ‘Gala’ in 2013 and during 223 DOY to 231 DOY for ‘Fuji’ in 2018. A preliminary report on the data collection for ‘Fuji’ in 2013 has been presented (Yang et al., 2019). Photosynthesis responses to intercellular CO_2_ concentration (*C*_i_) were based on 6–8 fully expanded leaves by modifying the ambient CO_2_ concentration (*C*_a_) in the leaf chamber in the following order: 400, 300, 200, 100, 50, 400, 600, 800, 1000, 1200, 1500, and 1800 μmol mol^-1^; the other parameters were fixed at 1500 μmol m^-2^ s^-1^ photosynthetic photon flux density (PPFD) using an integrated red-blue light source with 10% blue light, leaf temperatures at 30°C, and the vapor pressure deficit (VPD) between the leaf and air maintained below 1.5 kPa. The *R*_d_ was measured before sunrise (approximately 4:40–6:00). After the leaf was acclimated for 5–20 min per step to a steady state (changes in leaf stomatal conductance (*g*_sl_) less than ± 2% over 2 min), gas exchange was recorded. The *V*_cmax_ and *J*_max_ were estimated from the leaf net photosynthesis rate (*A*_l_)–*C*_i_ curve by fitting the biochemical model developed by Farquhar et al. (1980) and then were converted to values at 25°C based on parameters from Harley et al. (1992), which were obtained from barley chloroplasts (Nolan and Smillie, 1976). The spatial heterogeneity of *V*_cmax_, *J*_max_, and *R*_d_ within the canopy was predicted by linear functions depending on *N*_a_, which was predicted by a linear function of the daily cumulated PPFD (PPFD_d_). The PPFD_d_ was measured with light sensors S-LIA-M003 (Onset Computers, Bourne, MA, USA) on leaves located along a gradient of irradiance within the canopy. The PPFD_d_ in ‘Fuji’ was only measured in 2013.

Stomatal responses to PPFD, leaf temperature, and leaf surface VPD in the Jarvis model (Jarvis, 1976) were measured for a range of PPFDs (0–1500 μmol m^−2^ s^−1^), temperatures (20–35°C), and VPDs (1.0–3.5 kPa). During measurements, only one environmental factor was altered and the other parameters were fixed at 1500 μmol m^−2^ s^−1^ PPFD, leaf temperature at 30°C, and the VPD between the leaf and air was maintained below 1.5 kPa. Maximal stomatal conductance (*g_s_*_max_) was calculated based on the *g*_s_ values above the 90% percentile under optimal environmental conditions (800 ≤ PPFD ≤ 1500 μmol m^−2^ s^−1^, 20 ≤ T ≤ 30°C, 1.4 ≤ VPD ≤ 2 kPa, and *C*_a_ = 380 μmol mol^-1^) for all leaves. Leaf water use was represented by the leaf instantaneous *WUE* (*WUE*_l_) and was computed as the ratio of *A*_l_ to leaf transpiration rate (*E*_l_). Subsequently, the leaf area was measured on each leaf, and each leaf was oven-dried at 80°C to a constant weight and ground with a mortar. A subsample of 5.0 mg power was used to measure leaf nitrogen concentration using an automatic analyzer (FlowSys, Systea, Italy).

### Meteorological variables

Meteorological variables measured comprised incident PPFD, air temperature (*T*), air relative humidity (*RH*), and wind speed at 2 m above the ground in the orchard. Total PPFD was measured with a S-LIA-M003 sensor, air T and RH with a S-THB-M008 sensor, and wind speed with a S-WSB-M003 sensor (Onset Computers, Bourne, MA, USA). All data were averaged and stored at time steps of 1 min in a HOBO U30 data logger (Onset Computers). Inputs of incident radiation into RATP included atmospheric radiation, direct and diffuse PPFD, and near-infrared radiation (NIR). As only total PPFD was measured, atmosphere radiation was estimated in accordance with Brutsaert (1975), and diffuse/direct PPFD were estimated in accordance with Spitters et al. (1986) and Marco Bindi et al. (1992) based on measured climate data in an orchard. The NIR was assumed to have the same diffuse/direct ratio as PPFD. The number of directions of diffuse light interception computation was 46 and air CO_2_ concentration was 380 ppm during the simulation.

### Estimation of photosynthesis and transpiration at canopy scale

The canopy net daily photosynthesis rate per leaf area per day (*A*_c_, mmol m^−2^ d^−1^, equation 6) and transpiration rate per leaf area per day (*E*_c_, mol m^−2^ d^−1^, equation 7) were calculated by integrating the diurnal instantaneous canopy photosynthesis rate (*A*_h_, μmol m^−2^ s^−1^) and transpiration rate (*E*_h_, mmol m^−2^ s^−1^) which were simulated from isolated trees using the RATP model (available at http://www.openalea.gforge.inria.fr) (Pradal et al., 2008).


(6) $$A_{c}=\sum_{h=0.5}^{h=24}A_{h}\cdot 30\cdot 60\cdot 10^{-3}$$

(7) $$E_{c}=\sum_{h=0.5}^{h=24}E_{h}\cdot 30\cdot 60\cdot 10^{-3}$$

where 30 is the simulation step in 30 min and 10^−3^ is the unit conversion from μmol to mmol for *A*_c_ and from mmol to mol for *E*_c_. Canopy *WUE* (*WUE*_c_) was computed as the ratio of *A*_c_ to *E*_c_. The simulation was run from 190 DOY to 273 DOY, when all shoot growth had ceased, and then those days with PPFD_d_ more than 45 mol m^−2^ d^−1^ and less than 15 mol m^−2^ d^−1^ were classified as sunny and cloudy days, respectively. Key parameters used for the RATP model are listed in **Supplementary Table 1**.

### Analysis of virtual scenarios

The setting of virtual experiments was conducted as proposed by Massonnet et al. (2008) (**Table 1**). The simulation with the actual leaf distribution and functions for each treatment was the reference S0 scenario. The S1 scenario is that in which leaf functional parameters were switched between ‘Fuji’ and ‘Gala’ with the same interstock/rootstock combination. In the S2 scenario, leaf functional parameters for the same cultivar were switched between VV and VD. Starting with VD trees (S0a-b and S1e-f), the effect of foliage distribution on the two cultivars was evaluated by comparing S0a and S1e with S0b and S1f for ‘Gala’ and ‘Fuji’, respectively. To evaluate the effect of leaf functions on the two cultivars, S0a and S1f were compared with S0b and S1e for ‘Gala’ and ‘Fuji’ effects. Comparing S0c and S1g with S0d and S1h, and S0c and S1h to S0d and S1g, respectively, allowed us to perform similar analyses on VV trees. In the S2 scenario, the effects of interstocks were deciphered in a similar manner.

**Table 1:** Reference (S0) and switching scenarios (S1 and S2) obtained with the RATP model for ‘Fuji’ and ‘Gala’ apple trees grafted on vigorous rootstocks and associated with either a dwarf M26 interstock (VD) or a vigorous ‘Qinguan’ interstock (VV).

Scenario	Leaf distribution	Leaf function	Order of leaf distribution × function combination
S0	VD Fuji	VD Fuji	a
	VD Gala	VD Gala	b
	VV Fuji	VV Fuji	c
	VV Gala	VV Gala	d
S1	VD Fuji	VD Gala	e
	VD Gala	VD Fuji	f
	VV Fuji	VV Gala	g
	VV Gala	VV Fuji	h
S2	VD Fuji	VV Fuji	i
	VD Gala	VV Gala	j
	VV Fuji	VD Fuji	k
	VV Gala	VD Gala	l

### Statistical analyses and model validation

All data were analyzed using the R software (R Development Core Team, 2020). The linear and non-linear relationships between measurements and/or parameters were fitted with ‘*lm*’ and ‘*nls*’ functions, respectively. We performed linear modeling using the *lm*() function in R, accounting for variation in interstock, cultivar, and their interaction. The percent variance explained by each factor was calculated using the *anova*() function, and only those with a significant *P* value (<0.05) were visualized using the *ggplot2* package. After ANOVAs, significant differences among treatments were distinguished by the Duncan multiple mean comparison test at the *P*< 0.05 level using the *agricolae* package. To test if there were differences between interstocks, the ANCOVA for differences in the slope and intercept of linear relationships was performed.

The quality of the Farquhar and Jarvis sub-models’ parameterization was assessed by calculating the root mean square error (RMSE, equation 8) and relative root mean square error (RRMSE, equation 9), indicators of the overall relative accuracy of a model.


(8) $$RMSE=\sqrt{\frac{\sum_{i=1}^{n}{\left(O_{i}-S_{\text{i}}\right)}^{2}}{\text{n}}}$$

(9) $$RRMSE=\frac{RMSE}{\bar{O}}\times 100$$

where *O_i_* is the observed value and *S_i_* is the simulated value. The smaller the RMSE and RRMSE, the more accurate the simulation. In this study, model accuracy is considered excellent when RRMSE< 10%; good if 10% ≤ RRMSE< 20%; fair if 20% ≤ RRMSE< 30%; and poor if RRMSE ≥ 30% (Li et al., 2013).

## Results

### Leaf-scale photosynthesis, transpiration, and *WUE*

**Figure 1:**
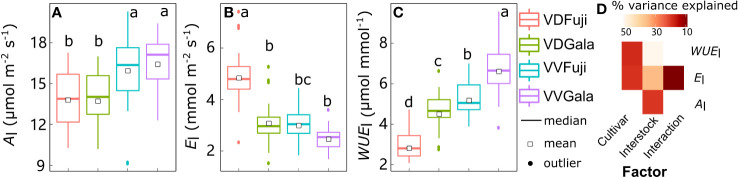
Boxplot of the leaf scale net photosynthesis rate (*A_l_*) **(A)**, transpiration (*E_l_*) **(B)**, and water use efficiency (*WUE*_l_) **(C)** in ‘Fuji’ and ‘Gala’ apple trees grafted on vigorous rootstocks associated either with a dwarf M26 interstock (VD) or a vigorous ‘Qinguan’ interstock (VV), and the corresponding amount of variance explained by cultivar, interstock, and their interactions **(D)**. The percent variance explained by each factor in the model is indicated using color for those factors which explain a significant portion of the variance (*p*< 0.05).

Although there was strong variability in *A*_l_, *E*_l_, and *WUE*_l_, the mean values were significantly affected by cultivar, interstock, and their interactions (**Figure 1**). Leaf *A*_l_ variation was significantly explained (20.7%) solely by interstock, with *A*_l_ higher in VV than in VD (+14.5% and +19.7% for ‘Fuji’ and ‘Gala’, respectively) (**Figures 1A, D**). The variation in *E*_l_ and *WUE*_l_ was significantly explained by both cultivar (18.5% for *E*_l_ and 18.7% for *WUE*_l_) and interstock (32.3% for *E*_l_ and 50.1% for *WUE*_l_), and their interaction significantly explained 5.67% of the variation for *E*_l_ (**Figure 1D**). The VD trees had higher *E_l_* in both ‘Fuji’ and ‘Gala’ (+60.4% and +24.4%) and lower *WUE_l_* in both ‘Fuji’ and ‘Gala’ (−45.6% and −31.4%) than VV trees (**Figures 1B, C**).

### Leaf stomatal conductance and responses to environmental factors

As responses of leaf *g*_s_/*g*_smax_ at leaf scale to environmental conditions and depending on interstocks were mostly consistent between cultivars for the Farquhar’s and Jarvis’ model parameters, results for ‘Gala’ are presented afterward, except when significant differences were found between cultivars.

**Figure 2:**
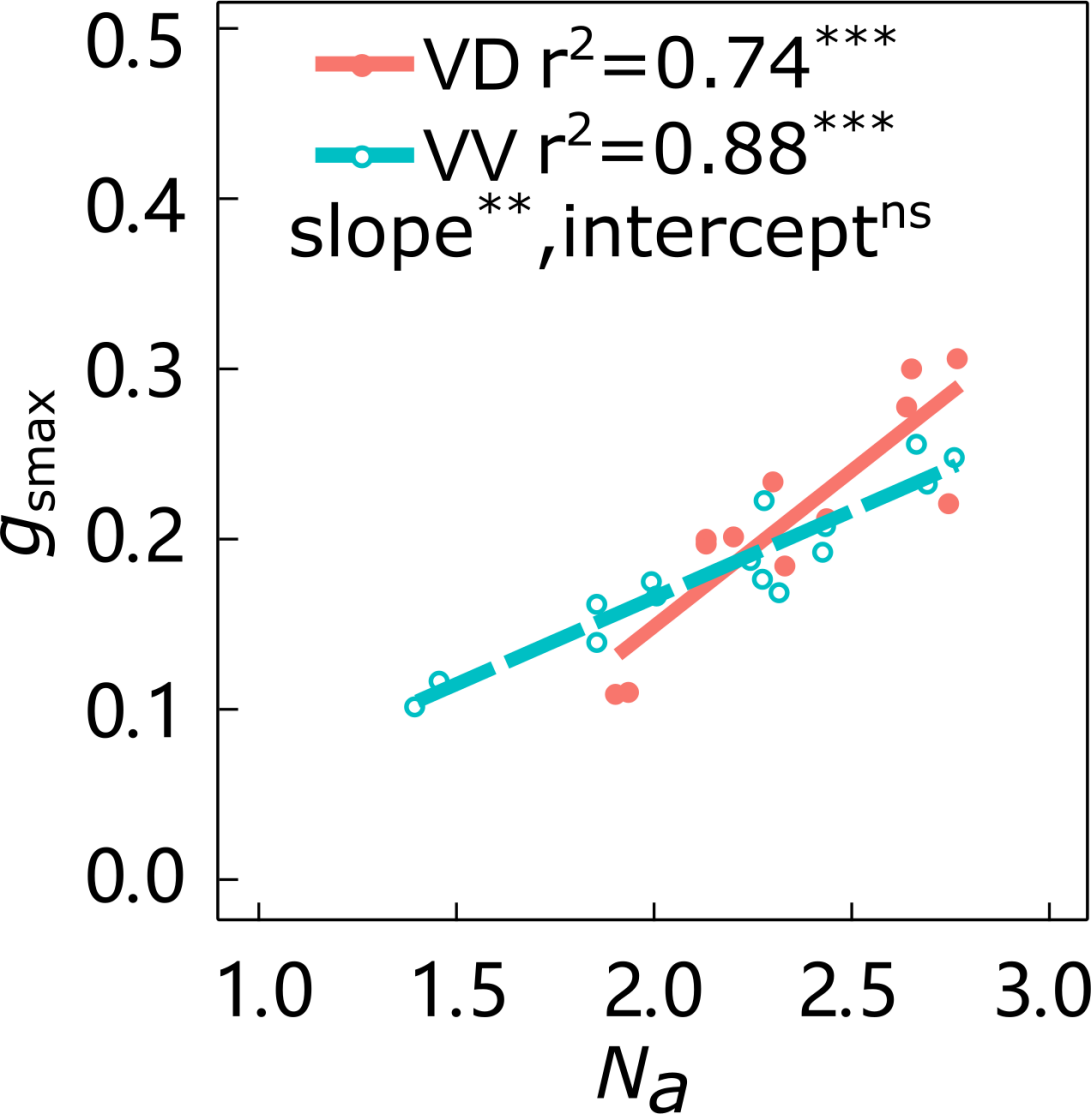
Relationships between leaf nitrogen content per area (*N*_a_) and maximum stomatal conductance (*g*_smax_) of ‘Gala’ apple trees grafted on vigorous rootstocks and associated with either a dwarf M26 interstock (VD) or a vigorous ‘Qinguan’ interstock (VV). Linear regression coefficients (r^2^) and significance for the regression slope and intercept are shown when *P* < 0.01 with ** and P < 0.001 with *** and ns means no significant difference between interstocks.

The slopes of the linear relationship between *g*_smax_ and *N*_a_ in ‘Gala’ and ‘Fuji’ were significantly affected by interstock variation, with VD trees having larger slopes than VV trees in both cultivars (**Figure 2**; **Supplementary Figure 1**). The effect of interstock on the intercept of the linear relationship between *g*_smax_ and *N*_a_ in ‘Gala’ was not significant, but it was significant for ‘Fuji’.

**Figure 3:**
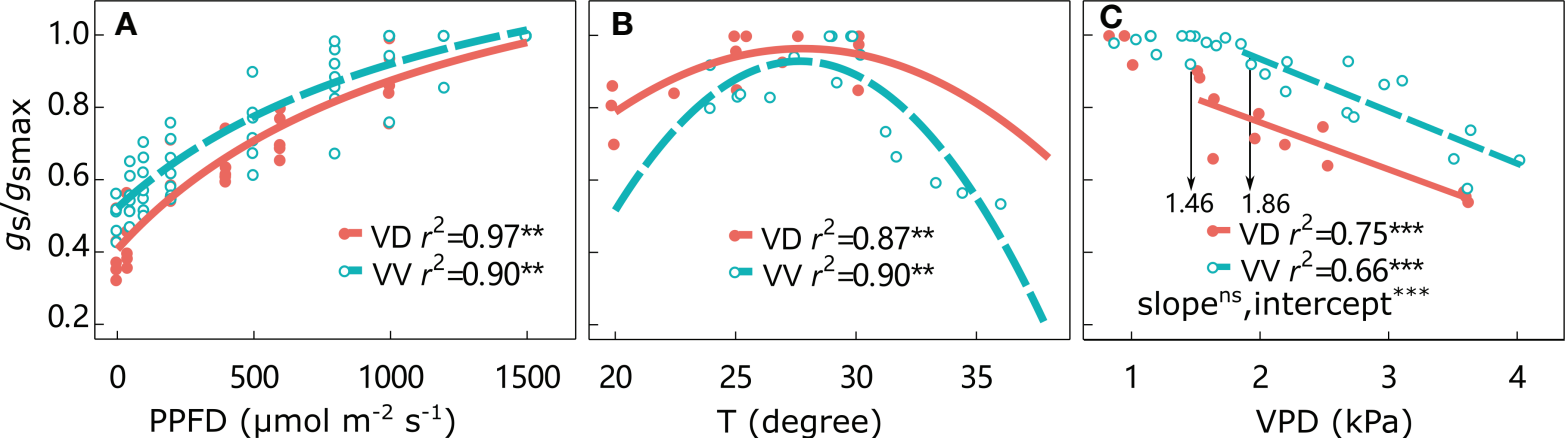
Relative stomatal conductance *g*_s_/*g*_smax_ responses to photosynthetically active radiation (PPFD) **(A)**, temperature (T) **(B)**, and water vapor pressure deficit (VPD) **(C)** for ‘Gala’ apple trees grafted on vigorous rootstocks and associated with either a dwarf M26 interstock (VD) or a vigorous ‘Qinguan’ interstock (VV) for the whole tree. The threshold VPD values ensuring *g*_s_ = *g*_smax_ are presented. Regression coefficients (r^2^) for all fitted lines, linear regression significance and interstock effect on a slope and intercept for VPD are shown when P < 0.01 with ** and P < 0.001 with ***.

The *g*_s_*/g*_smax_ responses to environmental factors are shown in **Figure 3** and **Supplementary Figure 2**. The *g*_s_*/g*_smax_ values continuously increased from 0 to the highest PPFD, irrespective of interstock. The optimal temperature was approximately 27°C for both VD- and VV-Gala, but higher values of 29.1°C for VD-Fuji and 31.8°C for VV-Fuji were found. The *g*_s_*/g*_smax_ remained high under increasing VPD until a threshold was attained. The VPD threshold was significantly higher in VV-Gala than in VD-Gala (1.46 and 1.86 kPa for VD-Gala and VV-Gala, respectively; *P*< 0.05). Beyond the threshold, *g*_s_*/g*_smax_ was significantly negatively correlated with VPD, irrespective of scion–interstock combinations. An ANCOVA test showed that there were significant differences in the regression intercepts between *g*_s_*/g*_smax_ with VPD for VV *vs*. VD, irrespective of cultivar, with higher *g*_s_*/g*_smax_ in VD than VV at the same point on the x-axis.

### Leaf nitrogen and *V*_cmax_, *J*_max_, and *R*_d_

**Figure 4:**
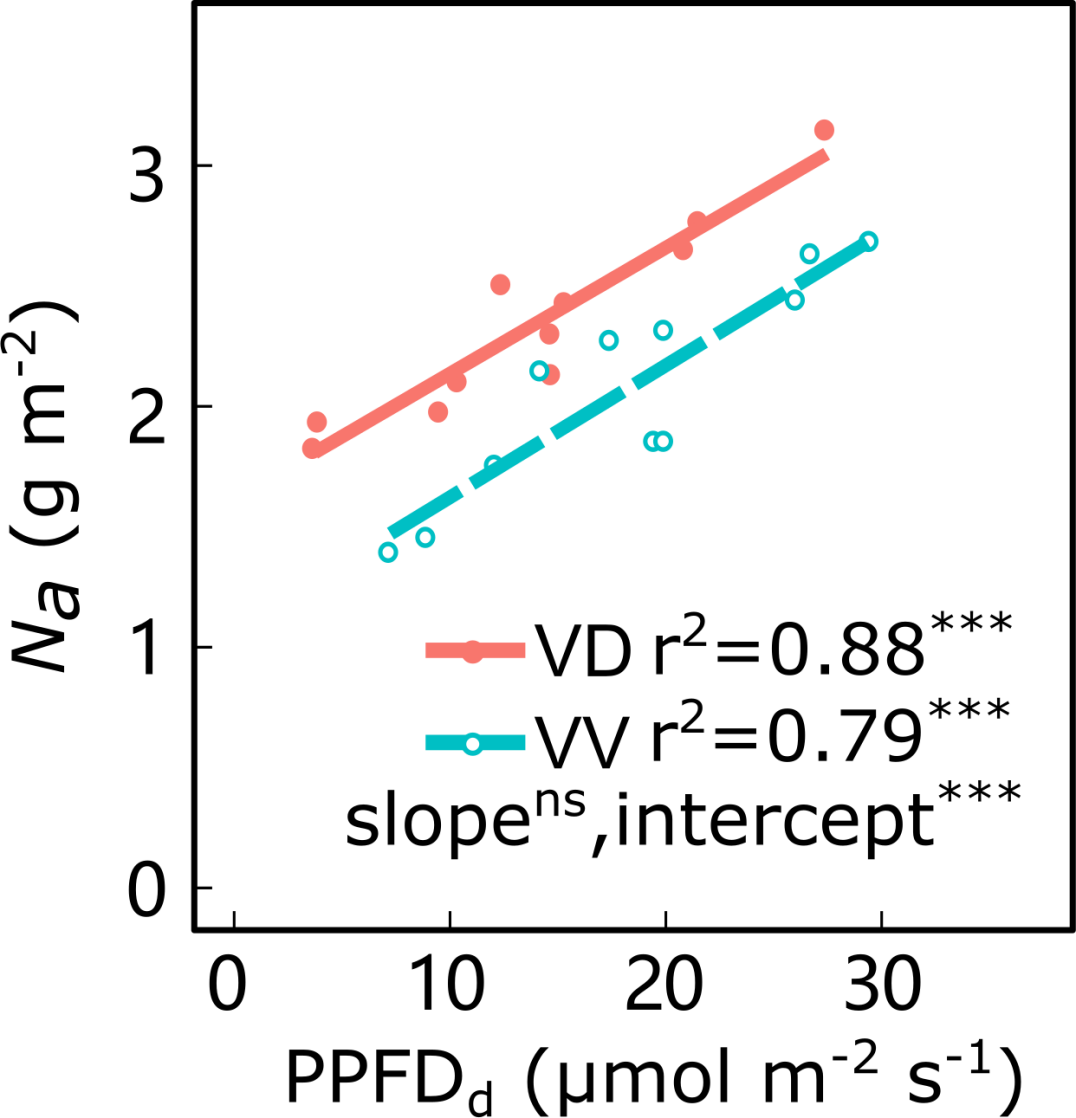
Relationships between daily cumulated photosynthetic photon flux density (PPFD_d_) and leaf nitrogen content per area (*N*_a_) for ‘Gala’ apple trees grafted on vigorous rootstocks associated either with a dwarf M26 interstock (VD) or a vigorous ‘Qinguan’ interstock (VV). Linear regression coefficients (r^2^) and significance and interstock effect on slope and intercept are shown when *P*< 0.001 with *** and no significant difference with ns.

Along the light gradient within the canopy, the leaf *N*_a_ decreased and was significantly positively correlated with the PPFD_d_, irrespective of cultivar and interstock (**Figure 4**; **Supplementary Figure 3**). An ANCOVA test showed that there were significant differences in intercepts between PPFD_d_ and *N*_a_ in both ‘Fuji’ and ‘Gala’, and in slopes for ‘Fuji’ for VV *vs*. VD trees. The VD trees had a greater *N*_a_ than VV for a given PPFD_d_, irrespective of cultivar, indicating that the VV had a greater reduction of *N*_a_ than VD through decreasing light.

**Figure 5:**
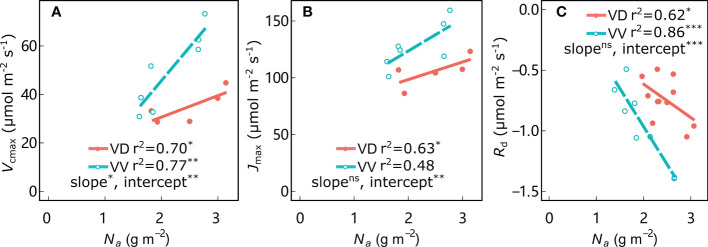
Relationships between maximum rates of carboxylation (*V*_cmax_) **(A)**, maximum rates of electron transport (*J*_max_) **(B)**, and dark respiration (*R*_d_) **(C)** with leaf nitrogen content per area (*N*_a_) for ‘Gala’ apple trees grafted on vigorous rootstocks associated with either a dwarf M26 interstock (VD) or a vigorous ‘Qinguan’ interstock (VV). Linear regression coefficients (r2) and significance and interstock effects on slopes and intercepts are shown by the level of significance of the p-values: *significant at 0.01 ≤ P< 0.05; **significant at 0.001 ≤ P< 0.01; ***significant at P< 0.001 and no significant difference with ns.

As expected, leaf *N*_a_ was significantly positively correlated with *V*_cmax_ and *J*_max_ in all treatments, and negatively correlated with *R*_d_ (**Figure 5**; **Supplementary Figure 4**). The regression intercepts between *V*_cmax_, *J*_max_, and *R*_d_ with *N*_a_ were significantly different for VV *vs*. VD in both ‘Fuji’ and ‘Gala’, indicating greater *V*_cmax_, *J*_max_ and *R*_d_ (in absolute values) in VV than in VD trees for a given *N*_a_ in both cultivars (except *J*_max_ for ‘Fuji’; **Supplementary Figure 4B**). In addition, a significantly different slope was observed only between *V*_cmax_ with *N*_a_ for VV *vs*. VD in ‘Gala’; the remainder of the corresponding regression slopes were not significantly different for VV *vs*. VD in both ‘Fuji’ and ‘Gala’, indicating higher sensitivity of *V*_cmax_ to *N*_a_ for VD than VV in ‘Gala’. Leaf mass per area (LMA) is a central trait within the leaf economics spectrum and is tightly coupled with leaf photosynthetic traits. The LMA was significantly positively correlated with PPFD_d_, irrespective of cultivar and interstock (**Supplementary Figure 5**). An ANCOVA test showed that there was a significant difference in slope between PPFD_d_ with LMA in ‘Fuji’ only for VV *vs*. VD trees. Interstock had no effect on the intercept of the linear relationship between PPFD_d_ with LMA in both ‘Fuji’ and ‘Gala’ for VV *vs*. VD trees.

### Canopy architecture, photosynthetic capacities, and *WUE*

**Figure 6:**
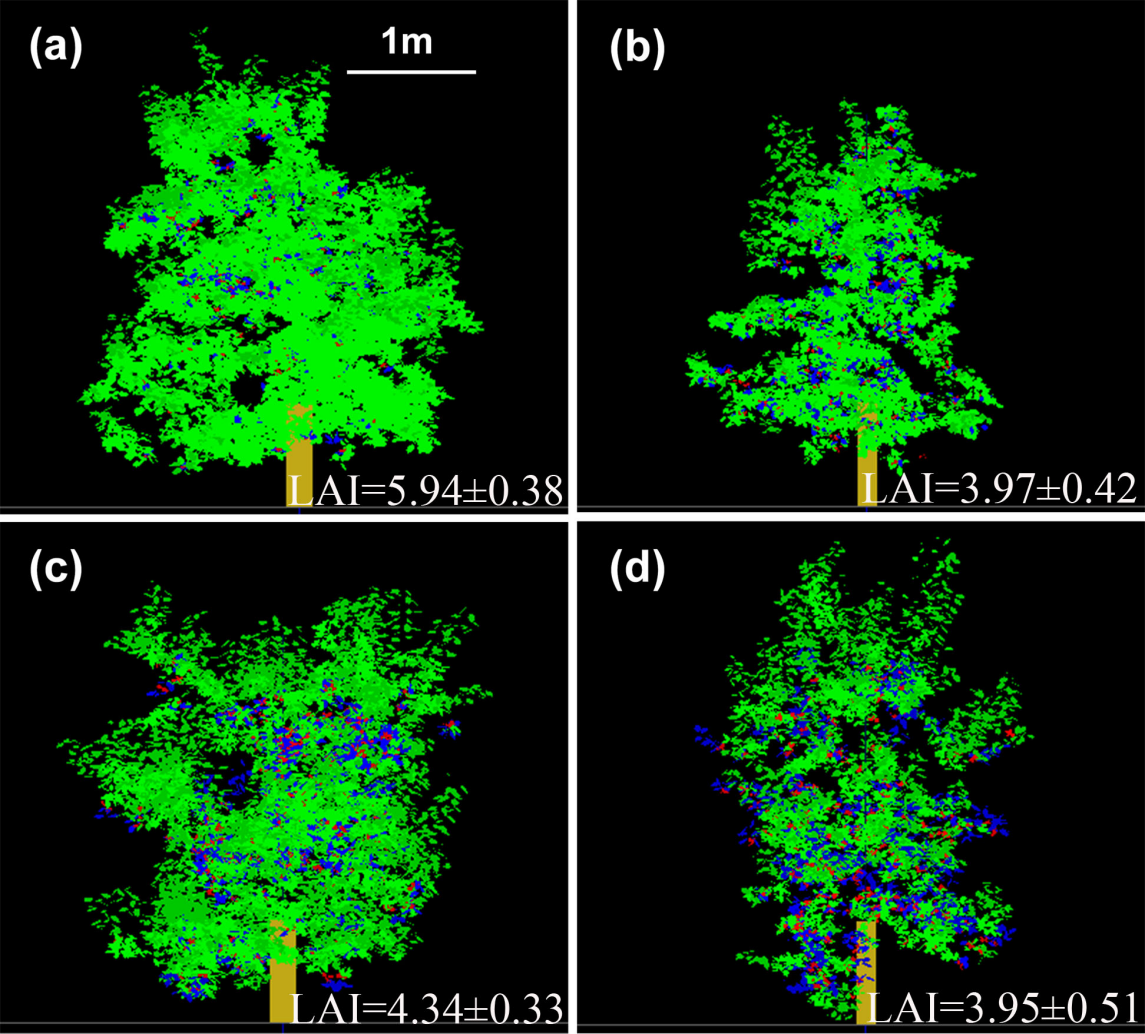
Three-dimensional representation of ‘Fuji’ **(A, B)** and ‘Gala’ **(C, D)** trees grafted on vigorous rootstocks associated with either a dwarf M26 interstock (VD, **(B, D)** or a vigorous ‘Qinguan’ interstock (VV, **(A, C)**. Leaf area index is given for each treatment. Virtual images were visualized with VegeSTAR software.

Leaf spatial distributions based on the 3D canopy were represented for ‘Fuji’ and ‘Gala’ in the two interstock/rootstock combinations (**Figure 6**). The LAI in VD trees was significantly lower (−33.2%) than in VV trees for ‘Fuji’ (*P*< 0.05), but were not significantly different between VD and VV trees for ‘Gala’. In addition, the tree vigor (represented by *TCSA*) in VD trees was significantly higher than that in VV trees (−37.1% and −47.0% for ‘Fuji’ and ‘Gala’, respectively), irrespective of the cultivar (**Supplementary Figure 6**).

**Figure 7:**
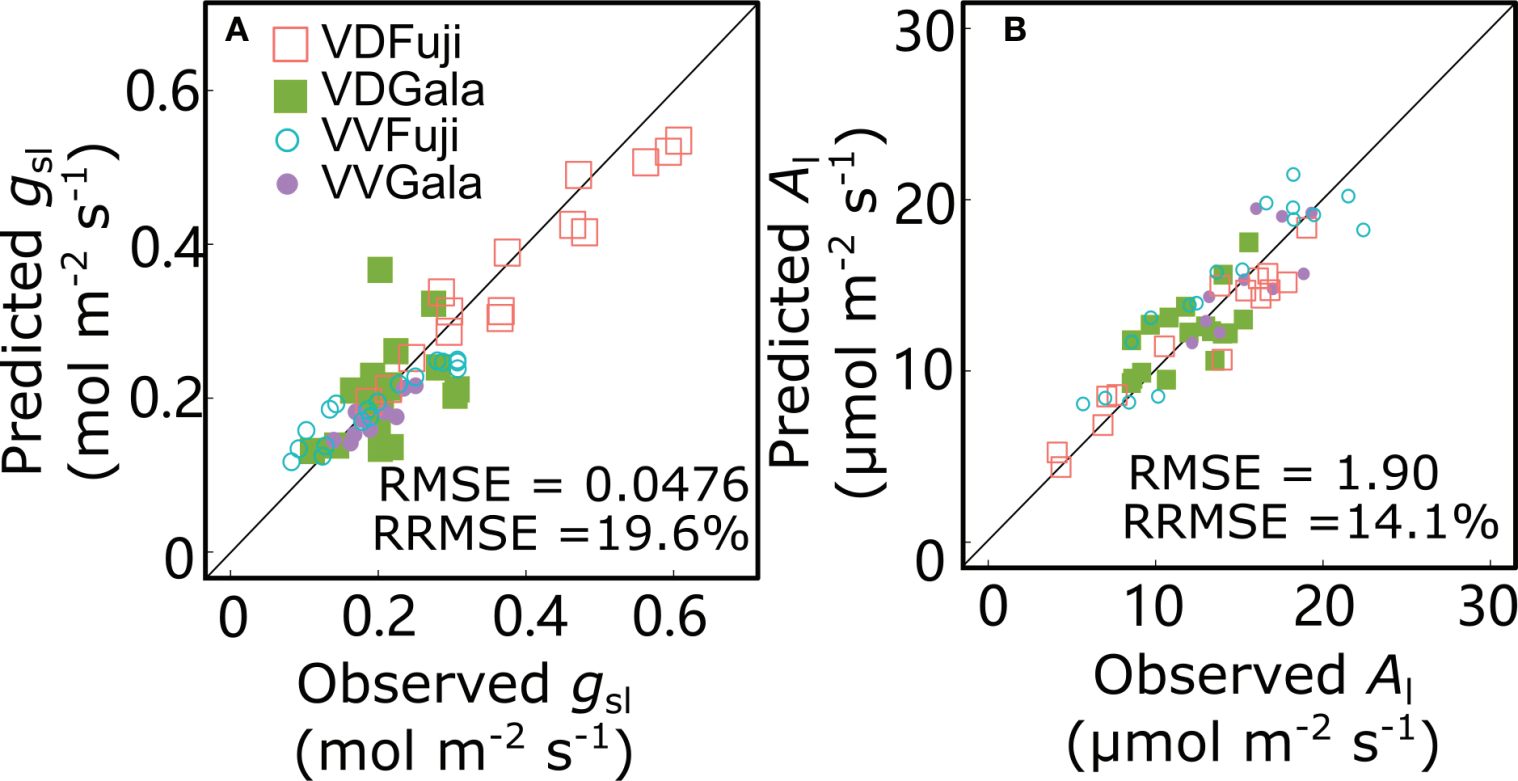
Relationships between observed and predicted stomatal conductance (*g*_sl_) **(A)** and net photosynthesis rate (*A*_l_) **(B)** for ‘Fuji’ and ‘Gala’ apple trees grafted on vigorous rootstocks associated with either a dwarf M26 interstock (VD) or a vigorous ‘Qinguan’ interstock (VV). Each point represents one measurement/simulation at the leaf level. The 1:1 lines are represented.

For the reference scenario (S0), the *g*_sl_ and *A*_l_ values predicted by the RATP model and the measured values were in good agreement with low RMSE (0.0476 mol m^−2^ s^−1^ and 1.90 μmol m^−2^ s^−1^ for *g*_sl_ and *A*_l_, respectively) and RRMSE (19.6% and 14.1% for *g*_sl_ and *A*_l_, respectively) values (**Figure 7**). Based on the of RRMSE values, the model showed good performance in predicting *g*_sl_ and *A*_l_.

**Figure 8:**
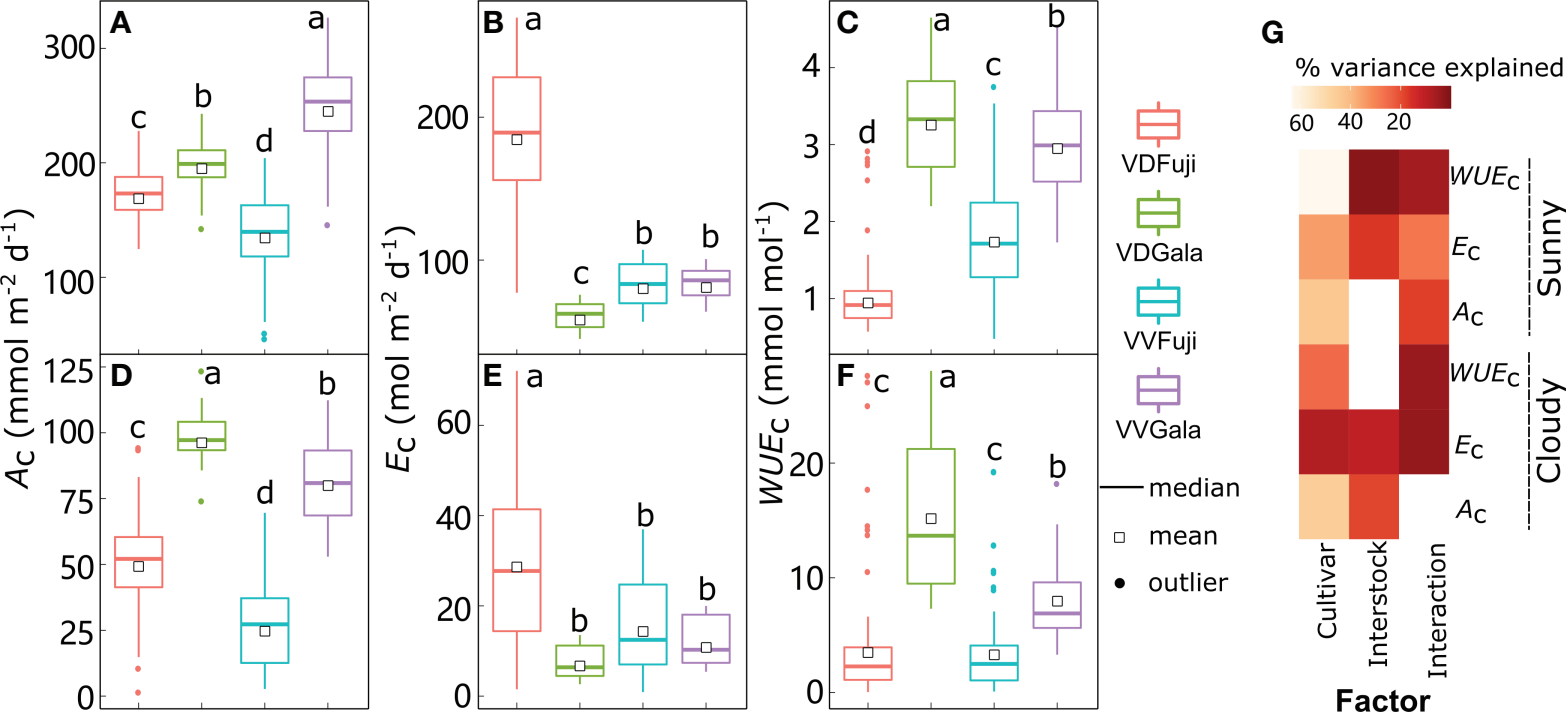
Boxplot of daily canopy net photosynthesis rate (*A*_c_) **(A, D)**, transpiration rate (*E*_c_) **(B, E)**, and water use efficiency (*WUE*_c_) **(C, F)** under sunny **(A–C)** and cloudy **(D–F)** conditions in ‘Fuji’ and ‘Gala’ apple trees grafted on a vigorous rootstock associated either with a dwarf M26 interstock (VD) or a vigorous ‘Qinguan’ interstock (VV), with corresponding amounts of variances in *A*_c_, *E*_c_ and *WUE*_c_ explained by cultivar, interstock, and their interaction **(G)**. The percent variance explained by each factor in the model is indicated using color for those factors that explain a significant portion of the variance (*p*< 0.05) and different letters indicate significant differences at *p*< 0.05.

Following the accurate predictions of leaf-scale gas exchange, the *A*_c_, *E*_c_, and *WUE*_c_ under both sunny and cloudy conditions were simulated with the RATP model (**Figure 8**). According to the estimations provided by the RATP model, variation in *A*_c_, *E*_c_, and *WUE*_c_ was significantly explained by cultivar, interstock, and their interactions (**Figure 8G**, except for the interstock effect on *A*_c_ under sunny conditions and *WUE*_c_ under cloudy conditions). Although the cultivar explained similar variation in *A*_c_ between sunny (43.6%) and cloudy (45.5%) conditions, variation in *E*_c_ and *WUE*_c_ explained by the cultivar decreased from 34.5% under sunny to 8.32% under cloudy conditions for *E*_c_ and from 63.1% under sunny to 24.7% under cloudy conditions for *WUE*_c_. In addition, interstock had a more significant influence on *A*_c_ under cloudy (18.9%) than sunny (0.058%) conditions.

Between cultivars grafted onto the same interstock, ‘Gala’ had significantly higher *A*_c_ and *WUE*_c_ than ‘Fuji’, irrespective of weather conditions (**Figures 8A, C, D, F**). Under sunny conditions, the *A*_c_ for ‘Fuji’ in VD trees was significantly greater (+24.7%) than that in VV trees, and this increased to +92.6% under cloudy conditions. Furthermore, the *E*_c_ for ‘Fuji’ was significantly higher (+125%) in VD trees than in VV trees, which decreased to +92.8% in cloudy conditions (**Figures 8B, E**). Thus, the *WUE*_c_ for ‘Fuji’ in VD was significantly lower (−44.0%) than that of VV trees in sunny conditions and decreased in cloudy conditions with VD, whereas VV trees had similar *WUE*_c_ (**Figures 8C, F**). For ‘Gala’, the *A*_c_ in VD trees was −19.5% and −27.3% less than VV under sunny conditions, and inversely, +19.9% larger than VV trees under cloudy conditions (**Figures 8A, D**). In addition, the *E*_c_ in VD−Gala was −27.3% less than that in VV-Gala under sunny conditions, and even smaller with VV trees under cloudy conditions (**Figures 8B, E**). Unsurprisingly, the VD−Gala had a significantly larger *WUE*_c_ than the VV−Gala (+11.3% under sunny conditions and +85.8% under cloudy conditions), irrespective of weather conditions (**Figures 8C, F**).

### Relative contributions of leaf function and leaf distribution on canopy photosynthetic capacities and *WUE*

**Figure 9:**
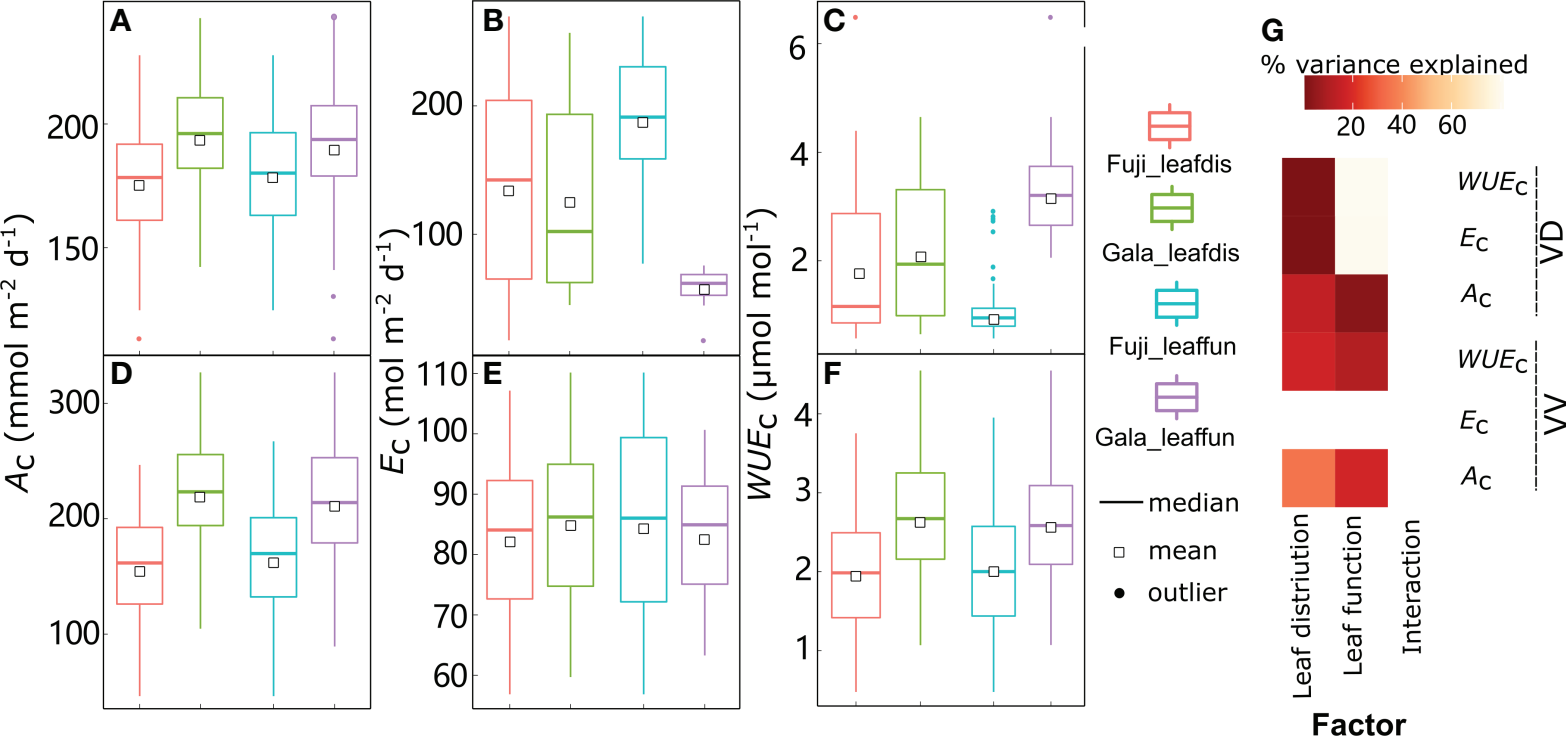
Boxplot of daily canopy net photosynthesis rate (*A*_c_) **(A, D)**, transpiration rate (*E*_c_) **(B, E)**, and water use efficiency (*WUE*_c_) **(C, F)** in dwarf interstock (VD) or vigorous interstock (VV) trees with switching cultivar leaf distribution (*leafdis*) and leaf function (*leaffun*) under sunny conditions, and corresponding amount of variances explained by cultivar *leafdis*, *leaffun* and their interaction **(G)**. The percent variance explained by each factor in the model is indicated using color for those factors that explain a significant portion of the variance (*p*< 0.05) and different letters indicate significant differences at *p*< 0.05.

**Figure 10:**
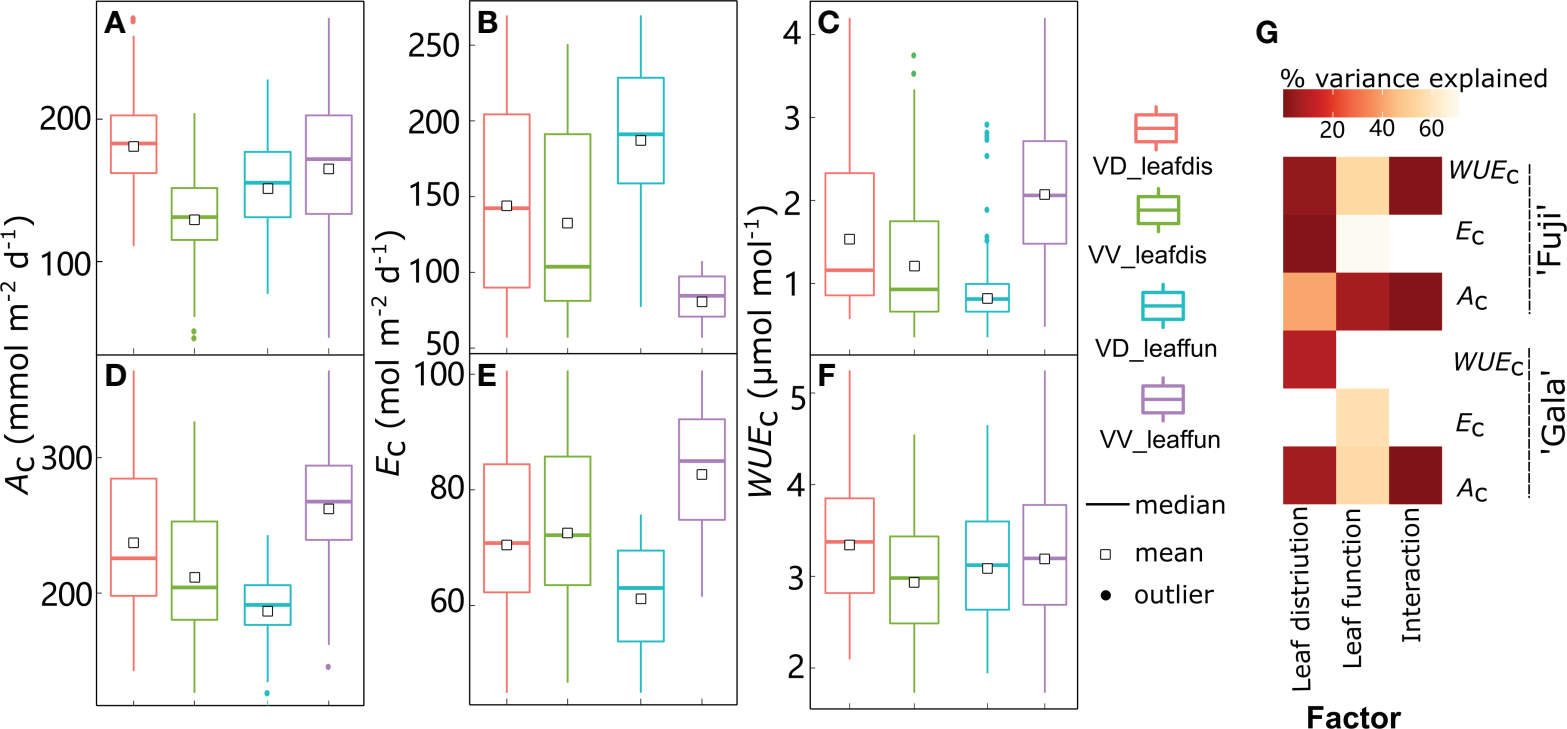
Boxplot of daily canopy net photosynthesis rate (*A*_c_) **(A, D)**, transpiration rate (*E*_c_) **(B, E)**, and water use efficiency (*WUE*_c_) **(C, F)** in ‘Fuji’ and ‘Gala’ trees with switching interstock (dwarf interstock (VD) and vigorous interstock (VV)) leaf distribution (*leafdis*) and leaf function (*leaffun*) under sunny conditions, and the corresponding amount of variance explained by interstock *leafdis*, *leaffun* and their interaction **(G)**. The percent variance explained by each factor in the model is indicated using color for those factors that explain a significant portion of the variance (*p*< 0.05) and different letters indicate significant differences at *p*< 0.05.

Scenario analyses showed that *A*_c_, *E*_c_, and *WUE*_c_ were modified by switching leaf distribution or functions compared with the reference S0 (**Figure 8**), but the amplitude of effects depended on the cultivar (**Figure 9** under sunny conditions and **Supplementary Figure 7** under cloudy conditions) or interstock (**Figure 10** under sunny conditions and **Supplementary Figure 8** under cloudy conditions).

By comparing scenarios S0 and S1, the relative roles of leaf distribution and functions were disentangled between ‘Gala’ and ‘Fuji’ in VV and VD trees under sunny conditions, respectively (**Figure 9**). Under sunny conditions, variation in *A*_c_ and *WUE*_c_ for both VV and VD trees and *E*_c_ for VD trees were significantly explained by both foliage distribution and function depending on the cultivar but without any interaction effects between cultivar leaf distribution and function, regardless of the variable considered to be significant (**Figure 9G**). Although variation in *E*_c_ was significantly explained by both leaf function (78.8%) and leaf distribution (0.37%) depending on the cultivar in VD trees, the latter contributed much less than the former. The *A*_c_ was significantly higher with ‘Gala’ leaf distribution (10.3% in VD and +40.6% in VV) or function (6.19% in VD and +29.4% in VV) compared with ‘Fuji’ (**Figures 9A, D**). In contrast, *E*_c_ was lower when considering ‘Gala’ rather than ‘Fuji’ leaf distribution (–6.51%) or functions (–68.2%), but this decrease was significant in VD trees only (**Figures 9B, E**). These resulted in notably higher *WUE*_c_ when considering ‘Gala’ leaf distribution (16.9% in VD and +34.0% in VV) or function (221% in VD and +27.2% in VV) than ‘Fuji’ (**Figures 9C, F**). Under cloudy conditions, both *A*_c_ (0.935–133 mmol m^−2^ d^−1^) and *E*_c_ (0.661–80.0 mol m^−2^ d^−1^) ranges showed significant decreases, whereas *WUE*_c_ (0.0117–36.4 mmol mol^-1^) ranges showed significant increases compared with sunny conditions (46.3–364 mmol m^−2^ d^−1^, 17.5–270 mol m^−2^ d^−1^ and 0.350–6.49 mmol mol^−1^ for *A*_c_, *E*_c_ and *WUE*_c_, respectively) (**Figure 9**, **Supplementary Figure 7**). Switching cultivar leaf distribution and function had consistent results under cloudy days, with the exception of the leaf distribution effect on *E*_c_ in VD trees, which was not significant but was significant under sunny days (**Supplemental Figure 7**). The percentage increases with ‘Gala’ *vs*. ‘Fuji’ leaf distribution in *A*_c_ on cloudy days were 3.54- and 2.37-fold greater than on sunny days, respectively, and the percentage increases with ‘Gala’ vs. ‘Fuji’ leaf function in *A*_c_ on cloudy days were 11.0- and 2.26-fold greater than on sunny days.

By comparing scenarios S0 and S2, the relative roles of leaf distribution and functions generated by VV and VD interstocks were disentangled in ‘Gala’ and ‘Fuji’ under sunny conditions, respectively (**Figure 10**). Under sunny conditions, interstock leaf distribution influenced *A*_c_ and *WUE*_c_,; VD trees showed significantly higher values in both cultivars, although the difference was more pronounced in ‘Fuji’ (+38.8% and 25.6% for *A*_c_ and *WUE*_c_, respectively) than ‘Gala’ (+11.9% and 13.7% for *A*_c_ and *WUE*_c_, respectively) (**Figures 10A, C, D, F**). Moreover, the leaf distribution owing to interstock explained a minor but significant 0.83% of the variance for *E*_c_ in ‘Fuji’ only, with VD trees showing 8.41% higher *E*_c_ than VV trees (**Figures 10B, E, G**). Regarding leaf functions, ‘Fuji’ trees with VV leaf functions had significantly higher *A*_c_ (+8.96%) and lower *E*_c_ (–56.0%), leading to significantly higher *WUE*_c_ (+143%) compared with VD leaf functions (**Figures 10A, B, C, G**). For ‘Gala’, trees with VV leaf functions had significantly higher *A*_c_ (39.0%) at the expense of higher *E*_c_ by a similar percentage (+34.8%) than trees with VD leaf functions, resulting in consistent *WUE*_c_ between VD and VV functions (**Figures 10D, E, F, G**). The interaction between leaf distribution and functions linked to interstock was significant for *A*_c_ and *WUE*_c_ in ‘Fuji’ and for *A*_c_ in ‘Gala’. Consistent with the results under sunny days, the distribution of VD leaves was more conductive to the improvement of *A*_c_ and *WUE*_c_ than VV leaf distribution (**Supplementary Figure 8**). More importantly, the percentage increase with VD over VV leaf distribution in *A*_c_ and *WUE*_c_ under cloudy days was 2.15- and 1.58-fold for ‘Fuji’, and 1.36- and 3.59-fold for those under sunny days.

## Discussion

### Interstocks affect leaf photosynthetic parameters through nitrogen distribution and partition

In the current study, we considered adult apple trees in fruiting years and discovered that the VD trees had lower leaf photosynthetic capacity than the VV trees, regardless of the cultivar. In parallel, significantly higher leaf *N*_a_ was observed in VD trees than in VV trees, consistent with previous results obtained under similar conditions (Aguirre et al., 2001; Fallahi et al., 2002; Samuolienė et al., 2016) and in other fruited trees, such as cherry (Gonçalves et al., 2006) and grapevine (Sharma et al., 2016).

Nitrogen is the primary component of nucleotides and proteins essential for plant photosynthesis, key enzymes, and acclimation to light across numerous species (Evans, 1989; Niinemets et al., 2015). In the present study, the relationships observed between *N*_a_ and PPFD_d_ and between *N*_a_ and photosynthetic parameters (*V*_cmax_, *J*_max_ and *R*_d_) (**Figures 4**, **5**) were consistent with previous studies (Le Roux et al., 2001; Walcroft et al., 2002; Massonnet et al., 2007; Niinemets et al., 2015) and in agreement with the optimization theory (Hirose and Werger, 1987). These relationships appeared robust and stable, regardless of the interstock. The range of *V*_cmax_ and *J*_max_ (**Figures 5**, **6**) was also consistent with results reported for apple trees in other regions (Cheng and Fuchigami, 2000; Greer, 2014), but the values were smaller than those reported by Massonnet et al. (2007), likely because of climatic differences or changes in tree development during the growing season (Walker et al., 2014).

To interpret the lower leaf photosynthetic capacity together with the higher leaf *N*_a_ content in VD trees than in VV trees, three assumptions can be proposed. First, the reduction of both *V*_cmax_ and *J*_max_ in VD trees (except for *J*_max_ in VD–Fuji trees) suggests that nitrogen partitioning into carboxylation (mainly by Rubisco) and bioenergetics (associated with electron transport) could be lower in VD than in VV trees. A second hypothesis could be that leaf nitrogen is in excess, which might reduce Rubisco activity, whereas nitrogen could serve as a storage protein (Cheng and Fuchigami, 2000). A third hypothesis is that trees grafted on a dwarf rootstock have impaired sensing of the balance between starch reserves and cellulose and hexose sugars, resulting in higher starch concentrations in the leaves. This has been demonstrated in ‘Royal Gala’ when grafted on dwarf M9 rootstock compared with a vigorous ‘Royal Gala’ rootstock (Foster et al., 2017). Consequently, leaves in trees with a dwarf interstock are expected to have higher starch content that, in turn, could inhibit their photosynthetic capacity *(* Wünsche et al., 2005*)*. However, disentangling these three assumptions or their possible combinations requires further investigation.

Moreover, *R*_d_ was smaller in VD trees than in VV trees, with values reported to be positively correlated with photosynthetic capacity, *N*_a_, and LMA, and proportional to *V*_cmax_ (Farquhar et al., 1980; Niinemets and Tenhunen, 1997). In the current case, the decrease of *R*_d_ in VD trees was associated with LMA in ‘Fuji’ but not in ‘Gala’, where LMA was not affected by interstock, nor with an increase of *N*_a_ as discussed above. We thus considered that the lower dark respiration was linked to the decrease in leaf biochemical potential (*V*_cmax_) in both cultivars and associated with LMA in ‘Fuji’. This reflects the different response of a cultivar to the same interstock/rootstock.

### Trees with vigorous interstocks appear less sensitive to abiotic stresses than dwarf interstock trees owing to slower stomata closure

The stomata adjust their aperture in response to environmental factors, leading to different tree *WUE*s. In the present study, we showed that dwarfing interstock induced faster stomatal closure when environmental conditions became stressful (i.e., high temperature, high VPD, and low light). In parallel, *g*_s_/*g*_smax_ remained higher in VV trees than in VD trees. The relationships between *g*_s_/*g*_smax_ and environmental factors were consistent with previous reports for apple (Dragoni et al., 2004; Massonnet et al., 2007) and walnut (Le Roux et al., 1999). This suggests that scions grafted on a vigorous interstock trees are able to maintain photosynthesis.

The *g*_sl_ and *E*_l_ in VD–Fuji were higher than in VV–Fuji (**Figure 1** and **Supplementary Figure 1**). However, dwarfing rootstocks may induce higher (Clearwater et al., 2004; Xu and Ediger, 2021) or lower *g*_sl_ and *E*_l_ (Higgs and Jones, 1990; Cohen and Naor, 2002) than vigorous rootstocks. Hydraulic resistance in different parts of the soil–root–graft union–stem–leaf continuum has been proposed to regulate water transport, leaf gas exchange, and dwarfing (Olien and Lakso, 1986; Higgs and Jones, 1990; Cohen and Naor, 2002; Cohen et al., 2007; Tworkoski and Fazio, 2015). In the current study, the scion was grafted on an interstock/rootstock combination. Thus, the differences in *g_s_*_l_ and *E*_l_ in ‘Fuji’ between interstocks may reflect differences in hydraulic architecture induced in the scion stem by the interstock/rootstock. However, given that such a difference was not observed in ‘Gala’, in which *g_s_*_l_ and *E*_l_ were similar between interstocks, this might be due to differences in the response between scion–interstock/rootstock (Baron et al., 2019) and measurement periods between the two cultivars. Indeed, leaf gas exchange was measured near harvest for ‘Gala’, when *g*_sl_ decreases significantly (Greer, 2019), whereas it was performed before harvest for ‘Fuji’. A modeling approach that integrates the responses of *g*_sl_ and *E*_l_ to root characteristics, hydraulic conductance, and chemical signals (Peccoux et al., 2018) would help to investigate the mechanisms of rootstock control on transpiration in apple trees.

At the leaf scale, *WUE*_l_ depends on both *A*_l_ and water loss through stomata. Indeed, higher stomatal conductance and photosynthesis are often followed by greater water use and carbon gain (Condon et al., 2004). Reduced *A*_l_ in both cultivars resulted in lower *WUE*_l_ in VD- rather than VV- trees. In ‘Fuji’ trees, this reduction was enhanced by higher *E*_l_ in trees with VD interstock. This suggests that the tradeoff between *A*_l_ and *g*_sl_ was altered by interstock but depends on the cultivar. In this case, the higher *g*_sl_ may be responsible for the higher yield potential in dwarfing apple trees, as observed in crop species (Blum, 2005).

Upscaling from leaf to canopy scale, VD–Gala had lower *E*_c_ than VV–Gala but their LAI (**Figure 6**) and light interception efficiency (Yang et al., 2016) were similar. This suggests that the lower *E*_c_ of VD–Gala than that of VV–Gala may result from the lower *E*_l_ at the leaf scale. Similarly, Cohen and Naor (2002) demonstrated that lower sap flow in dwarf than vigorous apple trees resulted from the leaf hydraulic conductance because the LAI between treatments was similar. However, the *E*_c_ of VD–Fuji was higher than that of VV–Fuji. Both the higher *E*_l_ and more favorable microclimate owing to lower LAI (**Figure 6**) and higher light interception efficiency (Yang et al., 2016) in VD–Fuji than VV–Fuji may contribute to the higher *E*_c_ of VD–Fuji than that of VV–Fuji. The virtual scenarios tested by switching interstock leaf distribution and function further dissected and verified that both VD leaf distribution and function enhanced *E*_c_ in ‘Fuji’. Moreover, the VV trees could maintain higher *g*_s_/*g*_smax_ under non-optimal environmental conditions, while not transpiring more water owing to their smaller *g*_sl_ compared with VD trees, irrespective of the cultivar. This led to higher *WUE* for VV trees and implied that water use was more conservative in vigorous interstock trees. This suggests that VV trees may be less sensitive to abiotic stress, possibly because of a stable leaf water status as suggested by previous studies (Liu et al., 2012; Bassett, 2013). In addition, ‘Gala’ leaf functions had a consistent effect on *A*_c_, *E*_c_ and *WUE*_c_, irrespective of the interstock or weather condition (**Figure 8**). However, the VD leaf functions had the opposite effect in ‘Fuji’ and ‘Gala’ (**Figure 10**). This suggests that cultivar leaf functions are independent of interstock, whereas interstock leaf functions depend on the cultivars.

### 3D architecture plays a crucial role in canopy carbon assimilation

Plant architecture has been reported to have a more profound effect on canopy photosynthesis at an early stage than at a stage with fully developed leaves in rice (Burgess et al., 2017). The simulations used steady-state leaf functions (such as *g*_sl_, *V*_cmax_ and *J*_max_) which are highly heterogeneous both temporally and spatially (Higgs and Jones, 1990; Ngao et al., 2017). In particular, the photosynthesis activity in leaves is higher at the beginning of the growing season (Greer, 2014). This could cause the photosynthesis activity in the canopy to be underestimated in the present work. However, the fully developed canopy, at which stage we measured leaf functions and architecture, was adequate to reveal differences among interstocks and cultivars.

In the reference scenario (S0), despite lower leaf-scale photosynthetic capacity in VD trees, *A*_c_ and *WUE*_c_ for ‘Fuji’ were significantly improved at the canopy scale in VD trees under both sunny and cloudy conditions, as well as for VD–Gala under cloudy conditions, when compared with the corresponding VV trees (**Figure 9**). It is likely that 3D architecture is responsible for higher *A*_c_ in VD trees through improved within-canopy light interception and micro-climate, which depend on weather conditions. In a previous study, we discovered that VD trees have a more even 3D leaf distribution, which leads to better light interception and deeper light penetration into the canopy vertically and horizontally than VV trees in both ‘Fuji’ and ‘Gala’ (Yang et al., 2016). Solar radiation is composed of a diffuse and a direct component; the fraction of diffuse light is higher on cloudy days than sunny days. Moreover, the light environment in VD trees for ‘Fuji’ is significantly improved for both diffuse and direct light and is only significantly improved for ‘Gala’ in VD trees for diffuse light compared with the corresponding VV trees (Yang et al., 2016; Yang et al., 2017). Thus, we consider that the more even 3D leaf distribution in VD trees led to more leaves receiving greater light and that this higher light interception had a positive effect on leaf *N_a_* (**Figure 4**), as suggested by the ‘optimization theory’ (Hirose and Werger, 1987). Therefore, the micro-climate around leaves (Woods et al., 2018) could be favorable and stimulate canopy photosynthetic potential in VD trees for ‘Fuji’ and ‘Gala’ under cloudy conditions.

Between interstocks, the leaf distribution of VD is more efficient than that of VV in improving *A*_c_ and *WUE*_c_ and similarly, the leaf distribution of ‘Gala’ is more efficient in improving *A*_c_ and *WUE*_c_ than ‘Fuji’ between cultivars owing to the reduced lower leaf clustering and within-tree self-shading (Yang et al., 2016). Thus, the relative frequency of leaves that are poorly illuminated is higher in VV than in VD and in ‘Fuji’ than in ‘Gala’, which results in higher dark respiration for maintenance. Furthermore, the positive effect of VD or ‘Gala’ leaf distribution on *A*_c_ and *WUE*_c_ was dissected and conformed by conducting a virtual scenario analysis (**Figures 9**, **10**). This is consistent with previous studies in which a LAI above the optimal value was shown to lead to decreased *A*_c_ (Anten et al., 1995; Song et al., 2020).

An additional point thing to note is that under cloudy conditions, the leaf distribution of VD and ‘Gala’, as well as the leaf function of ‘Gala’, were more efficient than under sunny conditions. However, diffuse light interception efficiency improved under the leaf distribution of VD and Gala more than direct (Yang et al., 2017). This could be because diffuse light generates a more homogeneous light distribution within the canopy than direct light and can avoid the light saturation constraint resulting from high light intensity (Gu et al., 2002) and allows plants to use diffuse light more efficiently than direct light (Urban et al., 2012; Li and Yang, 2015). In addition, changes in light components will alter the microclimate, such as air and soil temperature and VPD, and thereby directly or indirectly influence stomatal responses (Urban et al., 2012). However, further study is needed to disentangle the relative contributions among leaf nitrogen, photosynthetic parameters (e.g. *V*_cmax_, *J*_max_ and *R*_d_) and stomatal responses to environmental factors.

A tradeoff between *WUE* and yield is suspected, as increase in plant *WUE_c_* generally has been shown to result in reduced yield (Bassett, 2013). In the present study, the synchronous improvement of *A*_c_ and *WUE*_c_ indicated that selecting appropriate rootstock × scion combinations could suppress this tradeoff. Moreover, several genetic markers have been identified for dwarfing rootstocks (Foster et al., 2015) and also for tree architecture, light interception, and proxies of leaf stomatal conductance and transpiration (Virlet et al., 2015; Coupel-Ledru et al., 2022). This suggests potential genetic improvement of canopy performances through modification of both tree architecture and leaf functions could be considered.

## Conclusion

We showed that a dwarf interstock induced higher *g*_sl_ and transpiration in ‘Fuji’, and similar *g*_sl_ in dwarfed ‘Gala’ compared with those induced by a vigorous interstock. Dwarf interstock trees showed significantly lower leaf photosynthesis rate and *WUE* than vigorous trees. Canopy-scale simulations showed that lower leaf photosynthetic capacities and *WUE* were significantly improved in dwarf interstock trees compared with those of vigorous interstock trees. Switching scenarios highlighted that ‘Gala’ leaf functions and distribution and dwarf interstock leaf distributions enhanced canopy photosynthesis and *WUE* simultaneously, irrespective of weather conditions. This modeling approach paves the way to further optimize canopy photosynthetic capacity and *WUE* simultaneously through horticultural practices (e.g., rootstock and canopy management) that focus on both tree architecture and leaf functions.

## Data availability statement

The datasets presented in this study can be found in online repositories. The names of the repository/repositories and accession number(s) can be found below: http://doi.org/10.5281/zenodo.4049858.

## Author contributions

WY, XZ and DZ planned and designed the research. WY, XC and XZ performed experiments and conducted fieldwork. MS tested the model and provided related code. WY, XZ and EC implemented the models, analyzed the data, and wrote the manuscript. WY, XZ, EC, and MT reviewed and edited the manuscript. All authors contributed to the article and approved the submitted version.
