# Supplementary material for: Contributions of leaf distribution and leaf functions to photosynthesis and water-use efficiency from leaf to canopy in apple: A comparison of interstocks and cultivars
Source: Front Plant Sci. 2023 Apr 14;14:1117051. doi: 10.3389/fpls.2023.1117051 (PMC10146243; doi:10.3389/fpls.2023.1117051)
Supplement: Supplementary file 1 [file DataSheet_1.docx]

**Supplementary files**

**Table S1** Values, units and sources of parameters used for ‘Fuji’ and ‘Gala’ grafted on a vigorous rootstock associated either with a dwarf M26 interstock (VD) or a vigorous ‘Qinguan’ interstock (VV) in the RATP model. Parameter values were obtained either from literature, filed measurement, estimation and reconstruction based on experimental measurements or self-defined. Values with asterisk symbol (*) indicates that those are of the same among all treatments.

| Sub-models and functions | Parameters | Unit | Values | | | | Source |
| --- | --- | --- | --- | --- | --- | --- | --- |
|  |  |  | Fuji | | Gala | |  |
|  |  |  | VD | VV | VD | VV |  |
| leaf scale stomatal conductance sub-model of Jarvis(1976) | | | | | | | |
| *g*_smax_ = a*N*_a_+b | a_gmsx_ | mol s^-1^ g^-1^ | 0.222 | 0.0675 | 0.183 | 0.102 | Estimation |
|  | b_gmsx_ | mol m^-2^ s^-1^ | -0.172 | 0.0653 | -0.217 | -0.0377 |  |
| *g*_s_/*g*_smax_=(aPPFD+b)/(cPPFD+d) | a_PAR_ | μmol^-1^ m^2^ s | 0.004 | 0.003 | 0.009 | 0.0058 | Estimation |
|  | b_PAR_ | dimensionless | 3.67 | 3.90 | 3.13 | 2.78 |  |
|  | c_PAR_ | μmol^-1^ m^2^ s | 0.0014 | 0.002 | 0.0062 | 0.0040 |  |
|  | d_PAR_ | dimensionless | 6.63 | 6.43 | 7.68 | 5.33 |  |
| *g*_s_/*g*_smax_ = aT^2^+bT+c | a_T_ | °C^-2^ | -0.005 | -0.0038 | 0.0029 | -0.007 | Estimation |
|  | b_T_ | °C^-1^ | 0.291 | 0.242 | 0.161 | 0.388 |  |
|  | c_T_ | dimensionless | -3.29 | -2.81 | 1.27 | 4.44 |  |
| *g*_s_/*g*_smax_ = aVPD+b | a_VPD_ | kPa^-1^ | -0.182 | -0.178 | -0.130 | 0.149 | Estimation |
|  | b_VPD_ | dimensionless | 1.27 | 1.32 | 1.02 | 1.23 |  |
|  | VPD threshold | kPa | 1.8 | 2.0 | 1.46 | 1.86 |  |
| leaf scale photosynthesis sub-model of Farquhar (1976) | | | | | | | |
| *V*_cmax_ = a*N*_a_+b | a_Vcmax_ | μmol s^-1^ g^-1^ | 19.0 | 21.54 | 10.7 | 27.8 | Estimation |
|  | b_Vcmax_ | μmol m^-2^ s^-1^ | -8.35 | -2.59 | 8.77 | -9.85 |  |
| *J*_max_ = a*N*_a_+b | a_Jmax_ | μmol s^-1^ g^-1^ | 55.2 | 56.49 | 19.9 | 51.2 | Estimation |
|  | b_Jmax_ | μmol m^-2^ s^-1^ | -32.9 | -23.4 | 57.6 | 10.6 |  |
| *R*_d_ = a*N*_a_+b | a_Rd_ | μmol s^-1^ g^-1^ | -0.331 | -0.514 | 0.337 | 0.564 | Estimation |
|  | b_Rd_ | μmol m^-2^ s^-1^ | 0.314 | 0.359 | 0.0544 | 0.214 |  |
| leaf scale N concentration per unit area and daily culmulated PAR | | | | | | | |
| *N*_a_ = aPPPFD_d_+b | a_Na_ | g s mol^-1^ | 0.0537 | 0.0337 | 0.0523 | 0.0548 | Estimation in ‘Gala’ and Yang et al. (2019) for ‘Fuji’ |
|  | b_Na_ |  | 1.46 | 1.30 | 1.61 | 1.07 |  |
| Tree structure and grid | | | | | | | |
| Foligae distribution |  |  | Unique leaf 3D representation | | | | Reconstructed |
| Voxel size |  | m | 0.2* | | | | self- defined |
| Leaf optical properties | | | | | | | |
| Leaf absorbance in the PPFD |  | Proportion | 0.051* |  |  |  | Massonnet et al. (2008) |
| Leaf absorbance in the NIR |  | Proportion | 0.479* |  |  |  | Massonnet et al. (2008) |
| Soil optical properties | | | | | | | |
| Soil reflectance in the PPFD |  | Proportion | 0.075* |  |  |  | Sinoquet et al. (2001) |
| Soil reflectance in the NIR |  | Proportion | 0.2* |  |  |  | Sinoquet et al. (2001) |
| Relationship between leaf boundary layer conductance (gb，mm s^-1^) and wind speed (U, m s^-1^) | | | | | | | |
| gb = aU+b | a | mm m^-1^ | 10* |  |  |  | Daudet et al. (1999) |
|  | b | mm s^-1^ | 0.71* |  |  |  | Daudet et al. (1999) |
| Meteorological files | - | - | - | - | - | - | Filed measurement**^1^** |

**References**

Daudet, F.A., X. Le Roux, H. Sinoquet and B. Adam. (1999). Wind speed and leaf boundary layer conductance variation within tree crown: Consequences on leaf-to-atmosphere coupling and tree functions. *Agr. Forest Meteorol.* 97:171-185.

Massonnet, C., J. Regnard, P.É. Lauri, E. Costes and H. Sinoquet. (2008). Contributions of foliage distribution and leaf functions to light interception, transpiration and photosynthetic capacities in two apple cultivars at branch and tree scales. *Tree Physiol*. 28:665.

Sinoquet, H., X. Le Roux, B. Adam, T. Ameglio and F.A. Daudet. (2001). RATP: a model for simulating the spatial distribution of radiation absorption, transpiration and photosynthesis within canopies: application to an isolated tree crown. *Plant Cell Environ*. 24:395-406.

Yang, W., Zhang, X., Saudreau, M., Zhang, D., Costes, E., and Han, M. (2019). Photosynthetic capacity in 'Fuji' apple trees influenced by interstocks at leaf and canopy scale. *Acta. Hortic.* 1261, 77-84.


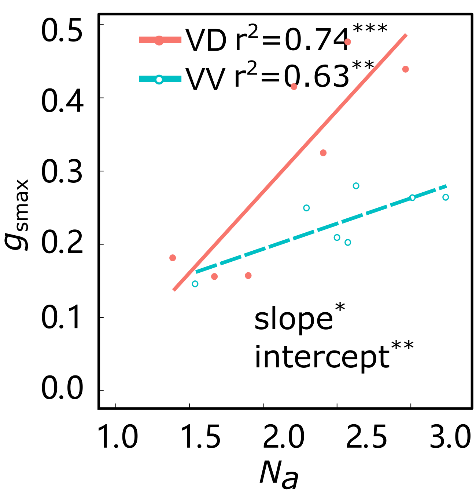


**Supplementary FIGURE 1 |** Relationships between leaf nitrogen content per area (*N*_a_) and maximum stomatal conductance (*g*_smax_) of ‘Fuji’ apple trees grafted on a vigorous rootstock and associated with either a dwarf M26 interstock (VD) or a vigorous ‘Qinguan’ interstock (VV). Linear regression coefficients (r^2^) and significance for the regression slope and intercept are shown when *P* < 0.01 with ** and P<0.001 with ***.


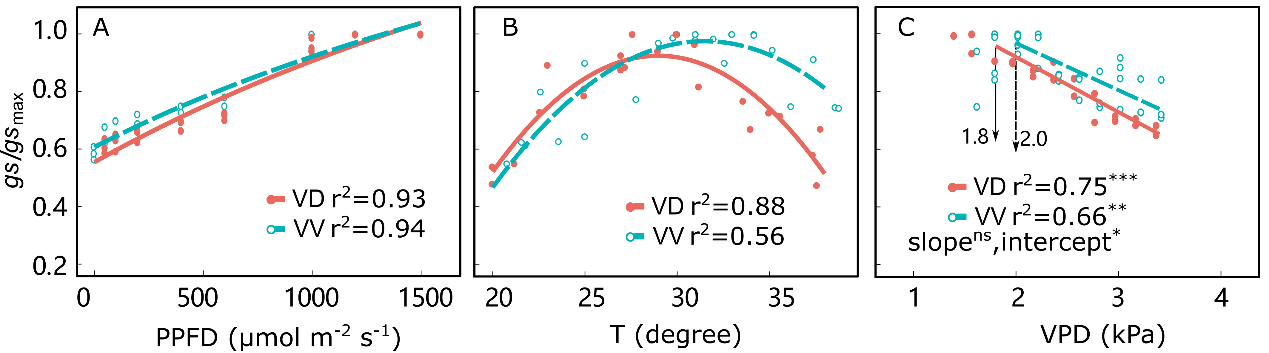


**Supplementary FIGURE 2 |** Relative stomatal conductance (*g*_s_*/g*_smax_) responses to photosynthetically active radiation (PPFD), temperature and water vapor pressure deficit (VPD) for ‘Fuji’ apple trees grafted on a vigorous rootstock and associated with either a dwarf M26 interstock (VD) or a vigorous ‘Qinguan’ interstock (VV) for the whole tree. The threshold VPD values ensuring *g_s_* = *g_smax_* are presented. Regression coefficients (r^2^) for all fitted lines, linear regression significance and interstock effect on slope and intercept for VPD are shown when *P* < 0.05 with *, P<0.001 with *** and ns mean no significant difference.


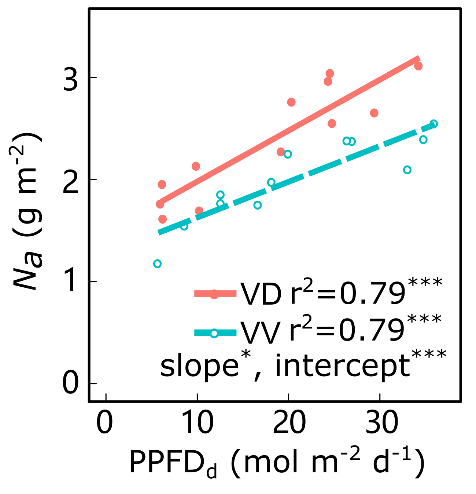


**Supplementary FIGURE 3 |** Relationships between daily cumulated photosynthetic photon ﬂux density (PPFD_d_) and leaf nitrogen content per area (*N*_a_) for ‘Fuji’ apple trees grafted on a vigorous rootstock associated either with a dwarf M26 interstock (VD) or a vigorous ‘Qinguan’ interstock (VV). Linear regression coefficients (r^2^) and significance and interstock effect on slope and interept are shown when P<0.05 with * and *P* < 0.001 with ***. Redraw from Yang et al. (2019).


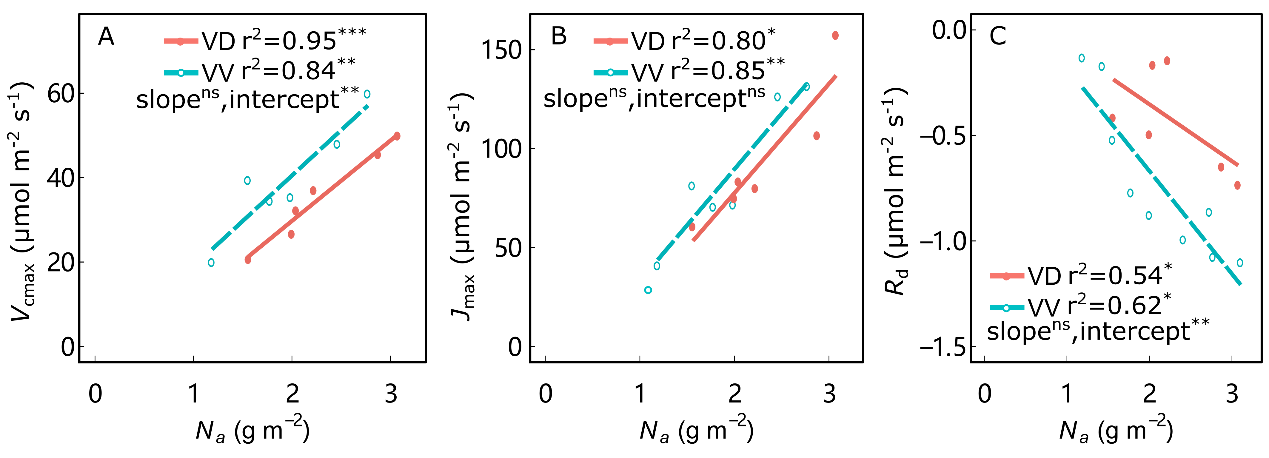


**Supplementary FIGURE 4 |** Relationships between maximum rate of carboxylation (*V*_cmax_, A), maximum rate of electron transport (*J*_max_*,* V) and dark respiration (*R*_d_, C) with leaf nitrogen content per area (*N*_a_) for ‘Fuji’ apple trees grafted on a vigorous rootstock associated with either a dwarf M26 interstock (VD) or a vigorous ‘Qinguan’ interstock (VV). Linear regression coefficients (r^2^) and significance and interstock effects on slopes and intercept are shown by the level of significance of the *p*-values: * significant at 0.01 ≤ *P* < 0.05; ** significant at 0.001 ≤ *P* < 0.01; ***significant at *P* < 0.001 and no significant difference with ns.


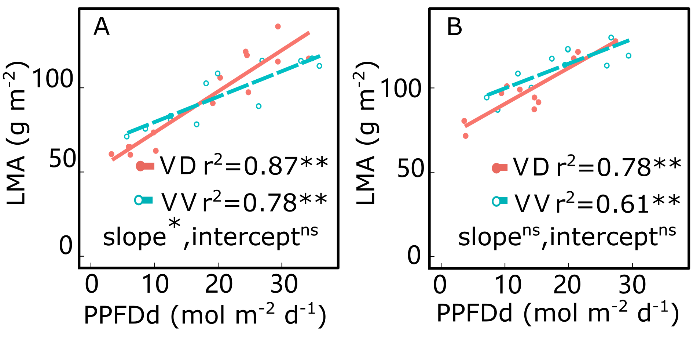


**Supplementary FIGURE 5 |** Relationships between daily cumulated photosynthetic photon ﬂux density (PPFD_d_) and leaf mass per area (LMA) in ‘Fuji’ (A) and ‘Gala’ (B) apple trees grafted on a vigorous rootstock associated with either a dwarf M26 interstock (VD) or a vigorous ‘Qinguan’ interstock (VV). Linear regression coefficients (r^2^) and significance and interstock effects on slopes and intercept are shown by the level of significance of the *p*-values: * significant at 0.01 ≤ *P* < 0.05; ** significant at 0.001 ≤ *P* < 0.01; ***significant at *P* < 0.001 and no significant difference with ns.


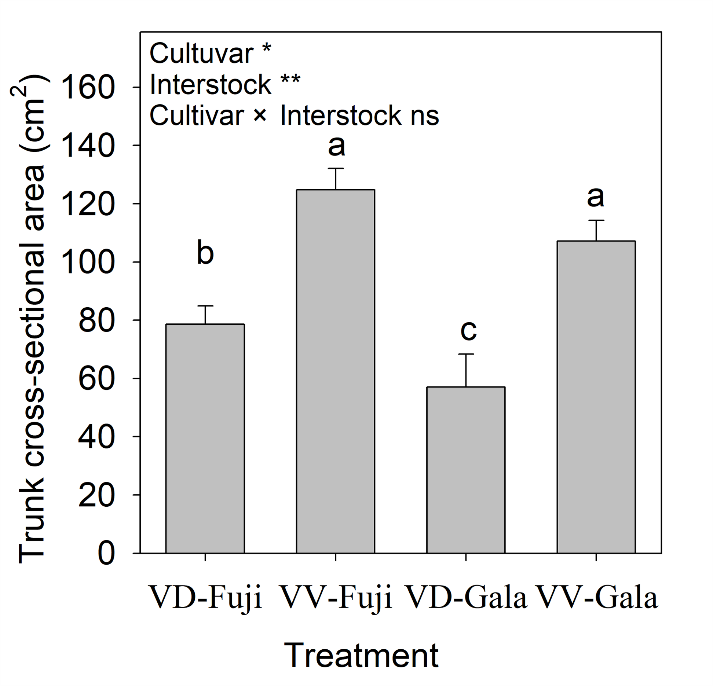


**Supplementary FIGURE 6 |** Mean trunk-cross sectional area (*TCSA*) of ‘Fuji’ and ‘Gala’ apple trees grafted on a vigorous rootstock and associated with either a dwarf M26 interstock (VD) or a vigorous ‘Qinguan’ interstock (VV). Effects of cultivar, interstock and their interactions were tested according to a two-way analysis of variance. When significant, the differences are indicated by the level of significance of the *P*-values: * significant at 0.01 ≤ *P* < 0.05; ** significant at 0.001 ≤ *P* < 0.01; ns, not significant. Different letters above bar indicate significant difference according to a Duncan's multiple range test at *P* < 0.05 level.

**
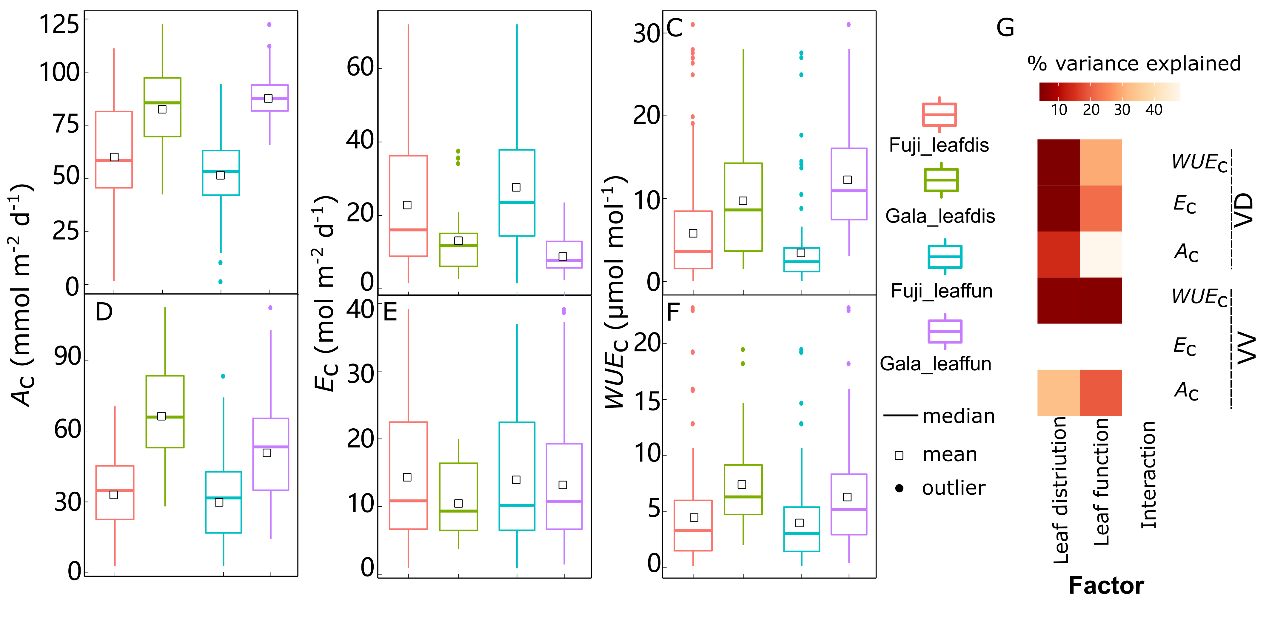
**

**Supplementary FIGURE 7 |** Boxplot of daily canopy net photosynthesis rate (*A*_c_, A and D), transpiration rate (*E*_c_, B and E) and water use efficiency (*WUE*_c_, C and F) in dwarf interstock (VD) or vigorous interstock (VV) trees with switching cultivar leaf distribution (*leafdis*) and leaf function (*leaffun*) under cloudy conditions, respectively, and corresponding amount of variances explained by cultivar *leafdis*, *leaffun* and their interaction (G). The percent variance explained by each factor in the model is indicated using color for those factors which explain a significant portion of the variance (*P* < 0.05) and different letters indicates significant differences at *P* < 0.05.


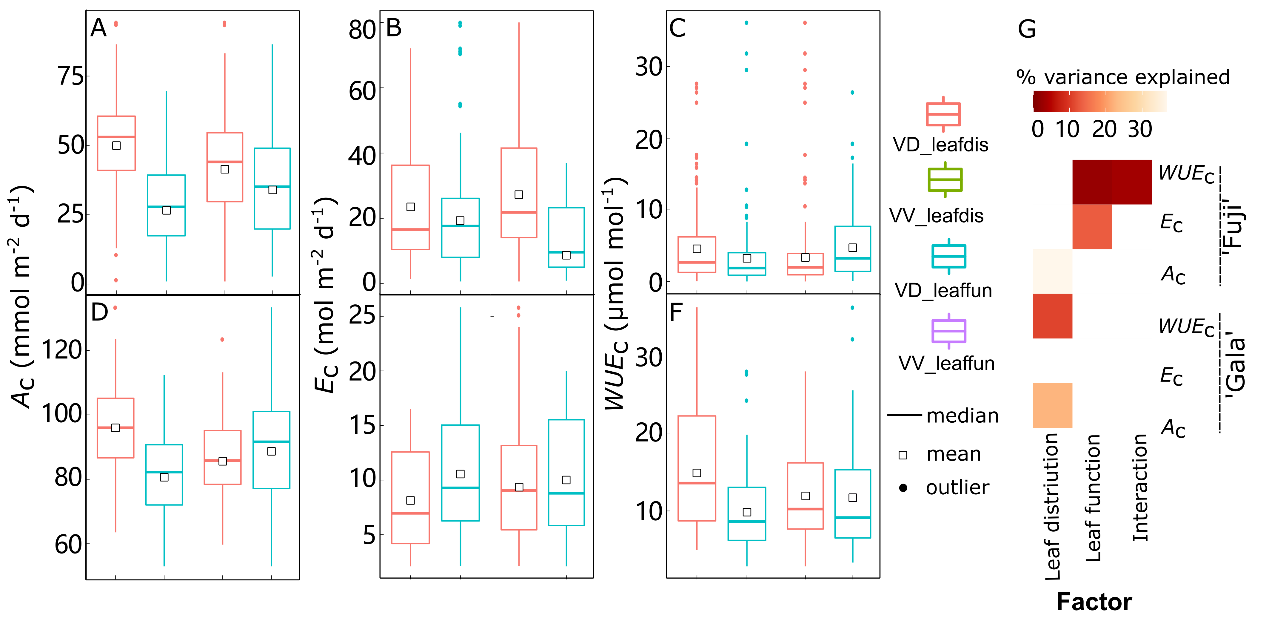


**Supplementary FIGURE 8 |** Boxplot of daily canopy net photosynthesis rate (*A*_c_, A and D), transpiration rate (*E*_c_, B and E) and water use efficiency (*WUE*_c_, C and F) in ‘Fuji’ and ‘Gala’ trees with switching interstock (dwarf interstock (VD) and vigorous interstock (VV)) leaf distribution (*leafdis*) and leaf function (*leaffun*) under cloudy conditions, and corresponding amount of variances explained by interstock *leafdis*, *leaffun* and their interaction (G). The percent variance explained by each factor in the model is indicated using color for those factors which explain a significant portion of the variance (*P* < 0.05) and different letters indicates significant differences at *P* < 0.05.
